# Single-cell analysis reveals inflammatory interactions driving macular degeneration

**DOI:** 10.1038/s41467-023-37025-7

**Published:** 2023-05-05

**Authors:** Manik Kuchroo, Marcello DiStasio, Eric Song, Eda Calapkulu, Le Zhang, Maryam Ige, Amar H. Sheth, Abdelilah Majdoubi, Madhvi Menon, Alexander Tong, Abhinav Godavarthi, Yu Xing, Scott Gigante, Holly Steach, Jessie Huang, Guillaume Huguet, Janhavi Narain, Kisung You, George Mourgkos, Rahul M. Dhodapkar, Matthew J. Hirn, Bastian Rieck, Guy Wolf, Smita Krishnaswamy, Brian P. Hafler

**Affiliations:** 1 Department of Neuroscience, Yale University, New Haven, CT USA; 2 Department of Pathology, Yale University, New Haven, CT USA; 3 Department of Ophthalmology and Visual Science, Yale University, New Haven, CT USA; 4 Department of Neurology, Yale University, New Haven, CT USA; 5 Yale School of Medicine, New Haven, CT USA; 6 Division of Infection, Immunity and Respiratory Medicine, University of Manchester, Manchester, UK; 7 Department of Computer Science, Yale University, New Haven, CT USA; 8 Department of Applied Math, Yale University, New Haven, CT USA; 9 Computational Biology, Bioinformatics Program, Yale University, New Haven, CT USA; 10 Department of Immunobiology, Yale University School of Medicine, New Haven, CT USA; 11 Mila—Quebec AI institute, Montréal, QC Canada; 12 Department of Mathematics and Statistics, Université de Montréal, Montréal, QC Canada; 13 Department of Computer Science, Rutgers University, New Brunswick, NJ USA; 14 Department of Genetics, Yale University, New Haven, CT USA; 15 Department of Computational Mathematics, Science and Engineering, Michigan State University, East Lansing, MI USA; 16 Department of Mathematics, Michigan State University, East Lansing, MI USA; 17 Department of Biosystems Science and Engineering, ETH Zurich, Zurich, Switzerland; 18 Broad Institute of MIT and Harvard, Cambridge, MA USA

**Keywords:** RNA sequencing, Macular degeneration, Molecular neuroscience, Diseases of the nervous system

## Abstract

Due to commonalities in pathophysiology, age-related macular degeneration (AMD) represents a uniquely accessible model to investigate therapies for neurodegenerative diseases, leading us to examine whether pathways of disease progression are shared across neurodegenerative conditions. Here we use single-nucleus RNA sequencing to profile lesions from 11 postmortem human retinas with age-related macular degeneration and 6 control retinas with no history of retinal disease. We create a machine-learning pipeline based on recent advances in data geometry and topology and identify activated glial populations enriched in the early phase of disease. Examining single-cell data from Alzheimer’s disease and progressive multiple sclerosis with our pipeline, we find a similar glial activation profile enriched in the early phase of these neurodegenerative diseases. In late-stage age-related macular degeneration, we identify a microglia-to-astrocyte signaling axis mediated by interleukin-1*β* which drives angiogenesis characteristic of disease pathogenesis. We validated this mechanism using in vitro and in vivo assays in mouse, identifying a possible new therapeutic target for AMD and possibly other neurodegenerative conditions. Thus, due to shared glial states, the retina provides a potential system for investigating therapeutic approaches in neurodegenerative diseases.

## Abstract (web summary)

Single-nucleus RNA-seq was used to profile 11 retinas with varying stages of age-related macular degeneration and 6 control retinas. The authors identified shared glial states across neurodegeneration, indicating that the retina provides a human system for investigating therapeutic approaches in neurodegeneration.

## Introduction

AMD is a neurodegenerative disease of the retina that affects 196 million individuals worldwide and has a significant impact on patient’s quality of life^1^. Similar to other neurodegenerative diseases of the central nervous system (CNS), such as Alzheimer’s disease (AD) and progressive multiple sclerosis (MS), AMD can be categorized into stages. Initially, in the early, ‘dry’ stage of AMD, extracellular amyloid-beta containing deposits known as drusen accumulate in the retina, leading to the activation of glia^2^. In advanced, ‘neovascular’ AMD, angiogenesis and fibrosis driven by vascular endothelial growth factor (VEGF) cause photoreceptor and vision loss^3^. In MS and AD, glial dysregulation is associated with neuronal damage and progressive neurologic impairment^4,5^. This raises the question of whether pathogenic glia activation states are shared across neurodegeneration, and whether the human retina can be used as a model for interventions targeting glial for similar neurodegenerative diseases.

While single-cell transcriptomics has given insight into the cellular perturbations in AD and MS^4–7^, a single-cell transcriptomic analysis of AMD has not been performed. To identify cell types and states enriched across stages of AMD, we performed massively parallel microfluidics-based single nucleus RNA-sequencing (snRNA-seq) to create a single-cell transcriptomic dataset of AMD pathology, comprising 70,973 cells across multiple stages of disease. In such large datasets, identifying cellular populations that drive disease and could be targeted for therapeutic benefit remains a challenge with current approaches. This often occurs because pathogenic populations may be a small subset of a recognized compartment of the tissue. Thus, it can be challenging to identify such populations among the noise and complexity present in single-cell data. To address this, we developed a topologically inspired machine-learning suite of tools called Cellular Analysis with Topology and Condensation Homology (CATCH). At the center of this framework is a pathogenic-population discovery pipeline whose key component is a method called diffusion condensation^8^. Diffusion condensation identifies groups of similar cells across scales systematically to discover subpopulations of interest within a data diffusion framework. In this approach, cells are iteratively pulled towards the weighted average of their neighbors in high-dimensional gene space, slowly eliminating variation. When cells come close to each other, diffusion condensation merges them together, creating a new cluster. When combined with a single-cell differential abundance method MELD^9^, diffusion condensation can identify distinct subpopulations associated with disease progression. This represents an improvement over clustering tools that partition the data based on metrics of cluster interconnectedness. Since this approach identifies specific disease-enriched populations, condition-specific signatures can be built and compared across neurodegenerative conditions, helping build a common understanding of shared disease mechanisms.

Using the CATCH pipeline, we identified two populations of activated glia, one microglial subset and one astrocyte subset, enriched in the early phase of dry AMD. These subsets were characterized by signatures of phagocytosis, lipid metabolism and lysosomal functions. By reapplying our pipeline to AD^4^ and MS^5^ single-cell datasets, we identified the same signatures in the early phases of multiple neurodegenerative diseases, indicating a common mechanism for glial activation in the early phase of neurodegeneration. The microglia and astrocyte expression signatures were validated in human retinal and brain tissue. In late-stage, neovascular AMD, CATCH identified an inflammasome expression signature in microglia as well as a pro-angiogenic signature in astrocytes. Through computational receptor-ligand interaction analysis, we identified a key signaling axis between microglia-derived IL-1*β* and pro-angiogenic astrocytes, the driver of neovascularization and photoreceptor loss in advanced disease in AMD^3^. Through a combination of human induced pluripotent stem cell (iPSC)-derived astrocyte stimulation assays, in vivo mouse experiments, and analysis of postmortem human AMD retinal samples, we validated this pro-angiogenic microglial-astrocyte axis mediated by IL-1*β* in late-stage neovascular AMD. As inflammasome and glial IL-1*β* signaling are important in AD and MS^10–12^, these pathways represent glial molecular signatures shared between neurodegenerative conditions that affect the retina and the brain. This study offers both a framework for identifying disease-affected cellular populations and disease signatures from complex single-cell data as well as key insights into the shared drivers of neurodegeneration.

## Results

### CATCH efficiently identifies, characterizes, and compares disease-enriched populations in complex single-cell transcriptomic data

**Fig. 1:**
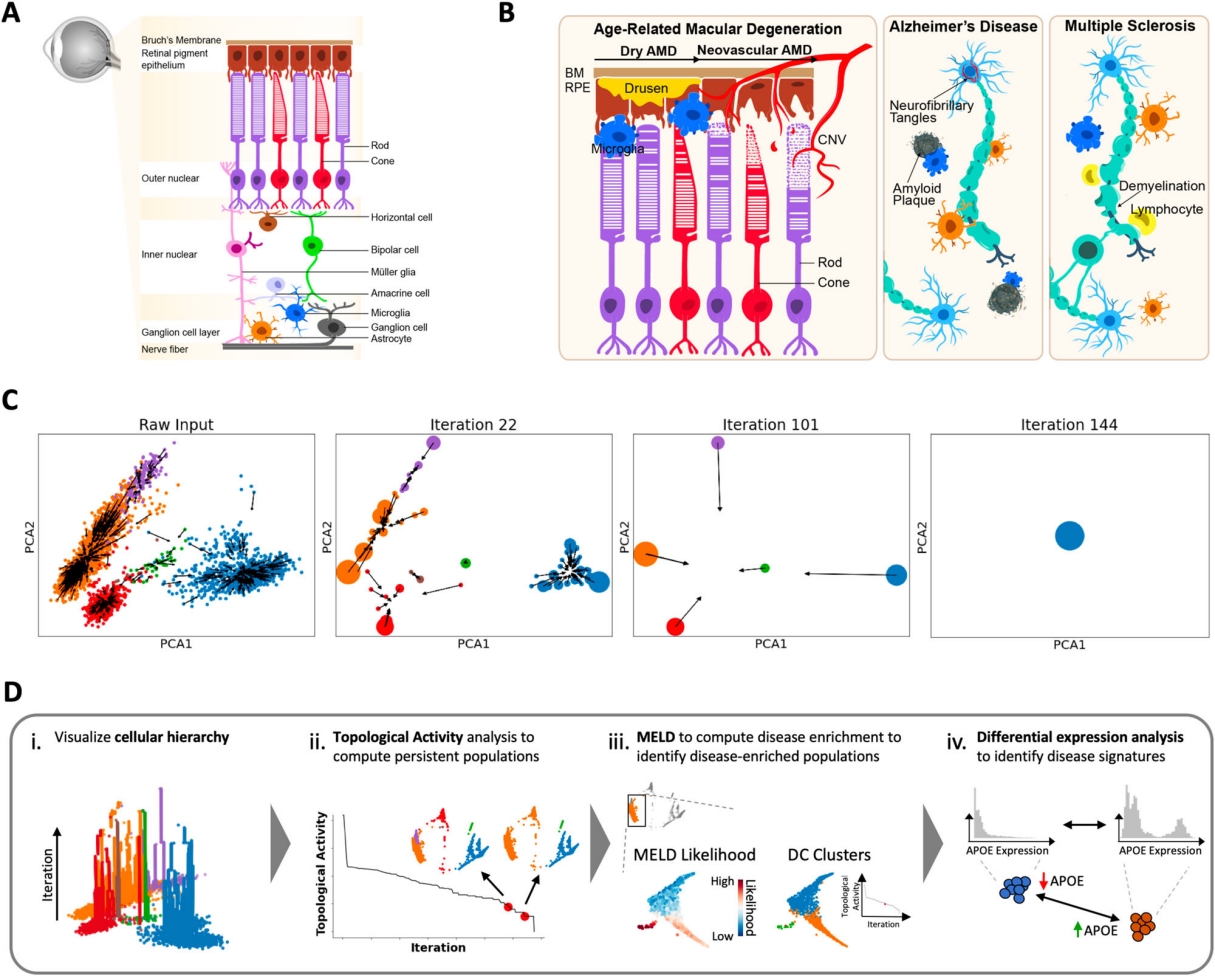
Overview of neurodegenerative disease processes and the topological diffusion condensation approach. **A** Sketch of retina cross-section showing layers and major cell types. **B** Illustration of the role of innate immune cells in neurodegenerative disease pathogenesis. In the dry stage of AMD, there is accumulation of extracellular drusen debris between Bruch’s membrane (BM) and the retinal pigment epithelium (RPE), leading to activation of glia. In the neovascular late-stage of AMD, VEGF-mediated choroidal neovascularization (CNV) develops, which can lead to vision loss through rod and cone photoreceptor cell death. Accumulation of extracellular plaques and intracellular neurofibrillary tangles in Alzheimer’s disease and myelin damage in progressive multiple sclerosis are both accompanied by microglia (blue) and astrocyte (orange) activation. **C** Visual description of cellular condensation process undertaken by diffusion condensation across four granularities. Points are moved to and merged with their nearest neighbors as determined by a weighted random walk over the data graph. Over many successive iterations, cells collapse, denoting cluster identity at various iterations. **D** The coarse graining process described in **C** creates hundreds of granularities of clusters, which can be analyzed in meaningful ways: (i) we can visualize the hierarchy of clusters computed by diffusion condensation, to identify the merging behavior across granularities; (ii) we can identify meaningful, persistent partitions of the data by performing topological activity analysis; (iii) in conjunction with MELD^9^, we can scan across these meaningful granularities to identify resolutions that optimally split disease-enriched populations of cells from healthy populations of cells and finally; (iv) we can compute differentially enriched genes between populations of interest.

As parts of the central nervous system (CNS), the retina contains many different functional layers and distinct strata that are occupied by a highly diverse set of cell types and states (Fig. 1A). Furthermore, as a component of the CNS, the retina shares features with the brain at the level of cell biology and degenerative pathology (Fig. 1B). Similar to AMD, MS and AD have defined disease phases, each with an early or acute active, and a late or chronic inactive disease stage^13–15^. To identify pathogenic cellular states enriched in AMD, and relate them to states found in AD and MS, we performed massively parallel microfluidics-based snRNA-seq to profile lesions from the macula of 11 retinas with varying degrees of AMD pathology and 6 control samples, creating a single-cell view of AMD pathology. We then applied a pipeline, CATCH, to parse this dataset into meaningful groupings of cell-types and states to identify pathogenic mechanisms of disease, which may be shared across neurodegenerative conditions. We used snRNA-seq for our analysis, which has been shown to perform well for sensitivity and cell-type classification as compared to scRNA-seq^16^. snRNA-seq has the added advantages that it minimizes gene expression changes resulting from tissue dissociation as well as minimizes challenges in dissociation for tissues such as the retina and brain

Cells can exist in various transcriptional states, which naturally fall into a hierarchy or organization. Within this hierarchy, cells of a more similar functional niche, for instance microglia and astrocytes, are more closely related to one another than cells of a more disparate niche, for instance microglia and endothelial cells. Learning this hierarchy from data is important to the development of a systematic understanding of biological function and can provide insight into mechanisms of disease pathogenesis. As cell types may be differentially affected by disease, the simultaneous identification and characterization of abundant classes of cells at coarse granularity as well as rare cell types or states at fine granularity provides a comprehensive framework for defining, modeling, and understanding specific cellular pathways in disease. While biological data has structure at many different levels of granularity, most clustering methods offer one or just a few levels of granularity. These few levels of granularity can create inaccurate identifications of disease-associated cellular states. To address this, we developed CATCH, a framework that combines the principles of data manifold geometry with computational topology to create a better understanding of cellular states across granularities. While the core component of CATCH, diffusion condensation^8^, and its mathematical properties^17^ have been established and used to identify multigranular structure in biomedical datasets^18^, it has not been applied to single-cell transcriptomic data. Here, we adapted and built a pipeline around diffusion condensation to systematically sweep through all possible granularities of the cellular hierarchy to identify pathogenic populations and infer mechanisms of neurodegeneration.

To learn the cellular hierarchy from complex single-cell transcriptomic data, we adapted diffusion condensation to efficiently move cells towards their most similar neighbors in terms of their transcriptomic profile across successive iterations. When cells collapse into one another, diffusion condensation merges them together, thereby clustering them at a specific level of granularity (Fig. 1C). By slowly condensing and then merging similar cells, diffusion condensation effectively learns how cells relate to one another over hundreds of levels of granularity. Since diffusion condensation does not force cells to merge at any given iteration, as done by other hierarchical clustering approaches, the length of time a cell, or cluster of merged cells, remains persistent denotes not only their transcriptomic interrelatedness but also their uniqueness from other cells. Cells that take only a few iterations to merge are very similar to one another, while cells that take a significant number of iterations to merge are more different in their overall transcriptomic profile. This approach is fundamentally separate from popular community detection clustering methods based metrics such as modularity and silhouette score, which optimize cluster labels based on network interconnectedness. Diffusion condensation is a coarse graining approach which slowly merges similar populations together across scales. This feature of the algorithm allows us to perform downstream analysis and identify populations enriched in disease states.

The CATCH framework utilizes the persistence characteristic of diffusion condensation to learn and analyze the cellular hierarchy to identify pathogenic transcriptomic states and to create robust signatures of disease from single-cell data. The cellular hierarchy is visualized to identify the hierarchical and persistence structure of the data (Fig. 1D-i). Meaningful granularities of the cellular hierarchy are identified through topological activity analysis, an analysis that identifies highly persistent and stable granularities for downstream characterization (Fig. 1D-ii). With this analysis, we identify clusters that isolate cells found disproportionately in pathogenic or healthy samples using the single-cell enrichment analysis method MELD^9^ (Fig. 1D-iii). Finally, we create rich signatures of disease by identifying differentially expressed genes in pathogenic populations of cells using a fast modification to Earth Mover’s Distance (EMD) that leverages the cellular hierarchy (Fig. 1D-iv).

For additional details on each component of the CATCH pipeline, including the adaptions to diffusion condensation, visualization of the cellular hierarchy, topological activity analysis and our implementation of differential expression analysis, see methods section.

#### Comparison to other clustering algorithms on synthetic and real single-cell data

We benchmarked our CATCH approach against existing clustering strategies applied to single-cell data. Using a combination of 40 synthetic single-cell datasets as well as real single-cell and flow cytometry data, we compared the clustering performance of our adapted implementation of diffusion condensation against Louvain and Leiden, multigranular clustering techniques often applied to single-cell data in packages in Monocle 3, as well as Seurat’s Shared Nearest Neighbors clustering algorithm and FlowSOM, state-of-art methods for clustering single-cell transcriptomic and flow cytometry data, respectively.

Splatter is a simulator of realistic single-cell data where ground truth cluster labels are known^19^. Using these ground truth labels, we generated increasingly noisy single-cell datasets with two different types of biological noise: variation and drop out (Supplementary Fig. 1A). With each of these datasets, we follow the CATCH framework: first we compute and visualize the condensation homology (Supplementary Fig. 1B) before performing topological activity analysis to identify the top four most persistent granularities (Supplementary Fig. 1C) and then finally computing adjusted rand index, a common measure for determining clustering accuracy against a set of ground truth cluster labels (Supplementary Fig. 1D), keeping the highest score from our comparisons. Intriguingly, the most persistent population (iv), nearly always had the highest adjusted rand index score. Using this comparison approach we compared diffusion condensation to Louvain, Leiden, and Seurat’s Shared Nearest Neighbors clustering algorithms across 40 synthetic single-cell datasets. For Louvain and leiden of the comparison approach, four different resolutions of clusters were computed and compared, keeping only the comparison, which produced the highest adjusted rand index. Across both increasing levels of drop out and increasing amounts of variation, CATCH performed better than Louvain, Leiden, and Seurat’s Shared Nearest Neighbors clustering algorithms across 10 different simulations. As noise increased to 0.7 and 0.9 drop out and 0.3 and 0.4 variation, CATCH out-performed other approaches in a statistically significant manner (*p* < 0.05, ANOVA with post hoc two-sided Student’s *t*-tests with multiple comparisons correction) (Supplementary Fig. 1E).

Next, we compared CATCH against Louvain and Leiden clustering approaches on real single-cell data where multigranular clusters had been identified by an biological expert^20,21^. First, we analyzed real single-cell transcriptomic data generated from a developing zebrafish with known cell-type cluster ground truths^20^. We organized these cluster labels into multigranular cluster labels by first aggregating 18 cell types found in four tissue types before aggregating them into three germ layers. In this manner, we produced ground truth cluster labels across granularities. We then compared the top four most persistent CATCH granularities against multigranular clusters computed using Louvain and Leiden, again tuning the resolution parameter to produce ten different cluster labels. At all granularities of ground truth cluster labels, CATCH out-performed Louvain and Leiden despite more granularities being computed for the comparison approaches (Supplementary Fig. 3B).

Finally, as flow cytometry gating analysis has long been held as the gold-standard for cell-type identification and comparison, we compared CATCH to other clustering approaches on flow cytometry data. Using 1.3 million cells generated from 30 patients, we compared the performance of CATCH to louvain, leiden and the flow cytometry clustering gold-standard FlowSOM^21^. Across all 30 comparisons, CATCH significantly out-performed other comparisons in a statistically significant way (two-sided *t*-test between CATCH and each of the other clustering approaches, *p*-value < 0.01) (Supplementary Fig. 3A). All of these comparisons establish that CATCH identifies known populations of cells in synthetic and real signal cell data better than established techniques, particularly when there is a high degree of biological noise and variation. Furthermore, CATCH computes a complete hierarchy of cellular states when identifying populations, allowing for subclustering groups of cells rapidly to identify activation states of interest. These subpopulation of cells are a direct subclustering of the coarser grain cluster of interest, allowing for comparison of cellular activation states. While one can repeatedly change parameters of other techniques to acquire finer or coarser grain clusters, these clusterings would be disconnected from one another, meaning a complete hierarchy is not captured and cellular groups across runs can shift dramatically. CATCH solves this problem by identifying clusterings across granularities within a single framework.

To further validate the computational analysis, we perform ablation studies on each component of the CATCH pipeline (Supplementary Fig. 2). Finally, we show the ability of this pipeline to identify rare cell types (Supplementary Fig. 5) and signatures of disease populations in real single-cell data (Supplementary Fig. 10). For an overview of computational analysis and additional comparisons, see methods section.

#### Single-nucleus RNA-seq analysis of the macula in human individuals with AMD pathology

**Fig. 2:**
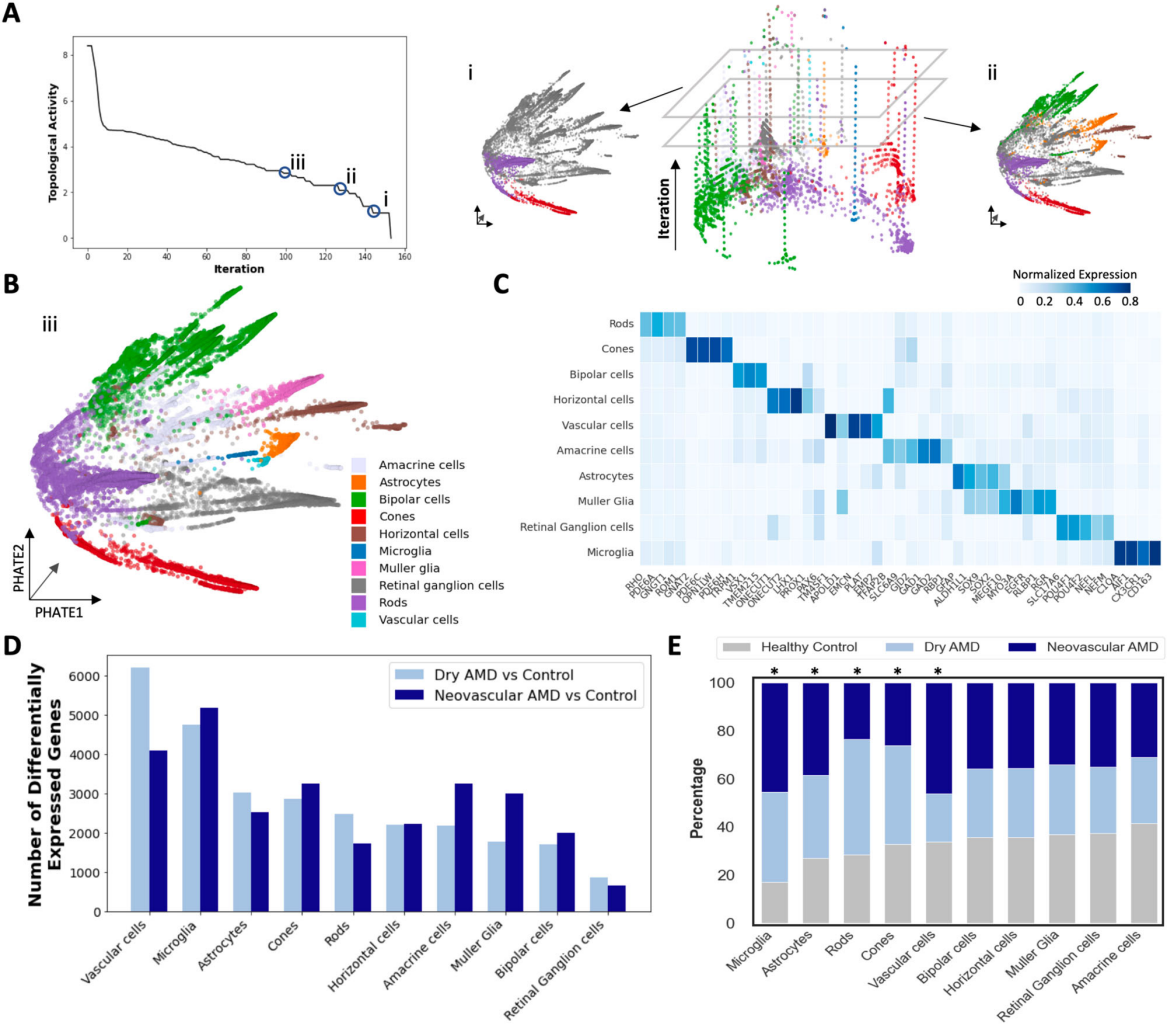
Single-nucleus RNA-seq profiling of the macula from human individuals with varying stages of AMD pathology. **A** (left) Topological activity analysis of human retina single-cell data across all condensation iterations. By computing gradients on topological activity (see Methods), we identify three granularities at which persistent partitions of the data occur (represented by resolutions i, ii and iii), and select them for downstream analysis. (right) Condensation process of AMD single-cell data visualized across iterations (from bottom to top) with the most coarse-grained granularity clusters visualized on PHATE embedding: resolution i. represents the most coarse-grained clusters and resolution ii. represents the second most coarse-grained clusters. **B** Populations identified at the finest granularity identified by topological activity analysis (resolution iii.) were visualized and all populations were assigned a cell type based on which cell-type gene signature they displayed the highest expression of. **C** Cell-type-specific genes visualized along with average normalized expression of known cell-type-specific marker genes. All major retinal cell types were identified by CATCH process described in **A**, **B**. **D** Differentially expressed genes identified by Wasserstein Earth Mover’s Distance (EMD) between cells from early-stage dry and late-stage neovascular AMD lesions and cells from control retinas on a cell-type-specific basis. Number of significantly differentially expressed genes between control and AMD cells reported in a cell type and stage-specific manner (FDR corrected *p*-value < 0.1). Cell types sorted by most differential genes between dry AMD and control comparison. Vascular cells, microglia and astrocytes have the most differentially expressed genes in dry AMD compared to control samples. **E** Bar chart indicates the contribution of cell types in each cluster from control, dry AMD and neovascular AMD samples. Microglia and astrocytes are the most statistically significantly enriched cell types in AMD, while rods and cones are the most depleted cell types in neovascular AMD. Vascular cells are the most enriched cell type in the neovascular AMD condition. All statistics were computed using two-sided multinomial tests with multiple comparisons correction (*p < 0.1).

We applied CATCH to the AMD snRNA-seq dataset to identify the major cell types present in the control and AMD samples. We performed topological activity analysis and identified three granularities of the cellular hierarchy for downstream analysis (granularities with low activity and high persistence). We visualized the snRNA-seq dataset using PHATE and the CATCH-defined clusters at the coarsest two identified granularities (Fig. 2A). When visualizing the third granularity, we observed a number of clusters, which we categorized as cell types based on the expression of previously established cell-type-specific marker genes^22^ (Supplementary Fig. 4A) (see Methods). Using this approach, we identified neuronal cell types, including retinal ganglion cells, horizontal cells, bipolar cells, rod photoreceptors, cone photoreceptors, and amacrine cells, as well as rare non-neuronal cell types, including microglia, astrocytes, Müller glia, and vascular cells (Fig. 2B, C). To determine if these populations could be found with established approaches, we applied Louvain^23^ clustering to the AMD single-cell data. Louvain revealed 22 populations at coarse granularity, and 40 populations at fine granularity (Supplementary Fig. 5A, B). Across both resolutions, however, rare innate immune cell types such as microglia, astrocytes and Müller glia, were not identified with the Louvain method, with markers specific for these cell types not localizing to any one cluster. Finally, to demonstrate the ability of CATCH to identify meaningful populations of cells across granularities, we further explored subtypes of bipolar cells, a diverse set of interneurons that transmits signals from rod and cone photoreceptors to retinal ganglion cells^24–26^. By analyzing a coarse granularity of the bipolar cells, we identified the first two major subtypes, ON-center and OFF-center (Supplementary Fig. 4B). By analyzing a finer granularity, we identified all 12 major subtypes of cells based on the expression of cell subtype-specific marker genes (Supplementary Fig. 4C–E).

To identify cell types implicated in AMD pathogenesis in an unbiased manner, we applied condensation-based differential expression analysis to the CATCH-identified cell types. By comparing the cells that originated from retinas with either dry or neovascular AMD to the cells from control retinas, we identified differentially expressed genes using Earth Mover’s Distance within each cell type (set FDR corrected *p*-value < 0.1 across all comparisons)^27^. By analyzing the number of differentially expressed genes across all cell types, we found that vascular cells, microglia, and astrocytes had the greatest number of differentially expressed genes across stages of AMD compared to control samples (Fig. 2D). Furthermore, we performed abundance analysis to identify if certain cell types were significantly more enriched in either dry or neovascular AMD. This analysis revealed a statistically significant increase in the proportion of microglia and astrocyte nuclei from donors with both dry and neovascular AMD compared to control samples (two-sided multinomial test, *p*-value < 0.01) (Fig. 2E). Furthermore, there was a statistically significant enrichment of vascular cells in neovascular AMD, highlighting the importance of vascular cells in the development of pathological angiogenesis present at that stage of disease (two-sided multinomial test, *p*-value < 0.01). There was a relative decrease in abundance of both rod and cone photoreceptors in advanced neovascular AMD, consistent with the known loss of photoreceptors in the advanced stage of disease (two-sided multinomial test, *p*-value < 0.01)(Fig. 2E). These findings suggest that non-neuronal cell types including microglia, astrocytes, and vascular cells are important cell types in AMD pathogenesis, with not only the most transcriptional alterations but also changes in abundance during AMD progression.

### Microglial activation signature identified in dry AMD is shared across the early phase of multiple neurodegenerative diseases

**Fig. 3:**
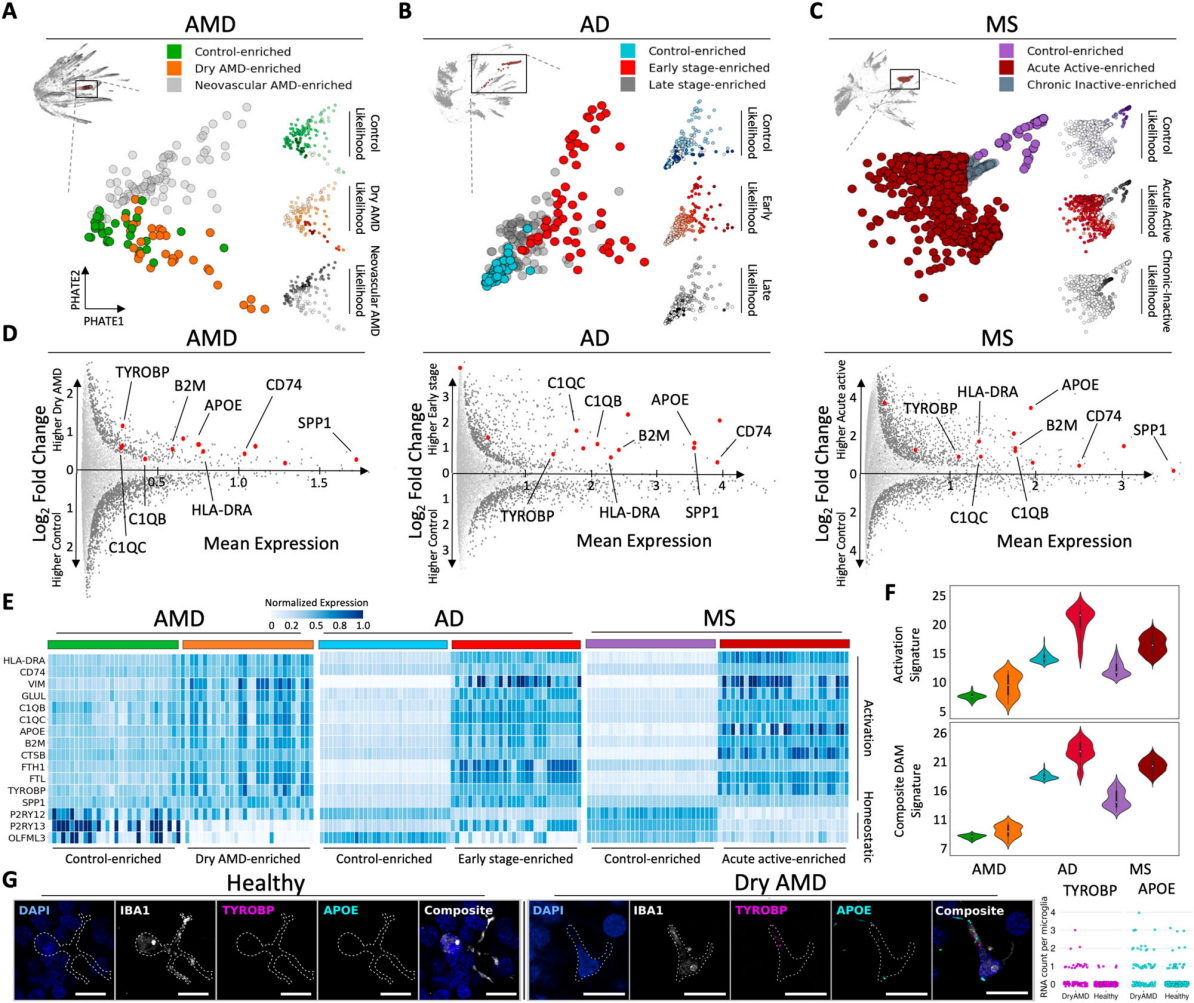
Fine grain analysis of microglia reveals a shared activation signature enriched in the early phase of three different neurodegenerative diseases. **A** 141 microglia identified by diffusion condensation at coarse granularity (upper left) can be further subdivided into three clusters at fine granularity, each enriched for cells from a different disease state. Disease state enrichment was calculated using MELD (right) for each condition: Control (top), dry AMD (middle) and neovascular AMD (bottom), with higher MELD likelihoods shown with darker colors. A resolution of the condensation homology, which optimally isolated MELD-likelihood scores from each condition was identified using topological activity analysis. Microglia are revisualized using PHATE. **B** As in panel **A**, three subsets of 288 microglia are found in AD with diffusion condensation and topological activity analysis, each enriched for cells from a different stage of pathology as computed by MELD (right). Cells are revisualized with PHATE. **C** As in panel **A**, three subsets of 1263 microglia are found in MS with diffusion condensation and topological activity analysis, each enriched for cells from a different stage of disease as computed by MELD (right). Cells are revisualized with PHATE. **D** Differential expression analysis between control-enriched and early or acute active disease-enriched microglia across neurodegenerative diseases reveals a shared activation pattern in early disease (increased expression of *TYROBP*, *B2M*, *APOE*, *CD74*, *SPP1*, *HLA-DR*, *C1QB*, *C1QC*). Significant differentially expressed genes are visualized in dark gray (two-sided EMD test with FDR corrected *p*-value < 0.1 as described in methods). **E** Heatmap demonstrating differences in expression of the neurodegenerative shared activation pattern and a homeostatic signature between control-enriched and early or acute active disease-enriched microglia across neurodegenerative diseases. Color conventions are as in panels **A**–**C**. Rows correspond to genes and columns represent individual cells. We have plotted 40 cells from each dataset selected through random sampling to reveal the difference between control-like and early disease-like cellular states. (**F, upper**) Composite microglial activation signature for the neurodegenerative shared activation pattern in control-enriched and early or acute active disease-enriched microglia across neurodegenerative diseases (*y*-axis—gene expression of signature). (**F, lower**) Disease-associated microglia (DAM) signature (from ref. ^7^) for control-enriched and early or acute active disease-enriched microglia across neurodegenerative diseases. Color conventions are as in panels **A**–**C** (*y*-axis—gene expression of signature). Details on statistics are available in methods section. **G** Micrographs of combined in situ RNA hybridization and IBA1 immunofluorescence demonstrating elevated expression of key components of the neurodegenerative shared activation pattern (*TYROBP* and *APOE*) in IBA1-positive cells, a marker of microglia, from retinas with dry AMD (right group) compared to control retinas (left group). All scale bars = 10 μm. The average number of puncta identified per IBA1-positive cell for *TYROBP* was 0.28 ± 0.05 in dry AMD (*n* = 191) vs. 0.02 ± 0.01 for control (*n* = 464; *p* < 1e-10; Chi-square test for 0 vs. >0). The average number of puncta identified per IBA1-positive cell for *APOE* was 0.57 ± 0.09 in dry AMD vs. 0.14 ± 0.03 for control (*p* < 1e-08; Chi-square test for 0 vs. >0).

While microglia activation states and their dynamics have been identified in mouse models of AD^7^ and related expression states found in humans^28^, it is not well understood to what extent these states and dynamics are shared across human neurodegenerative diseases. The study of microglia in the CNS has been difficult due to their rarity, requiring focused enrichment strategies^7,28^. With the ability of CATCH to sweep across all hierarchies of clusters, we can identify subpopulations of rare cell types at fine granularity to perform a rigorous and in-depth analysis of cellular states. To identify microglial subpopulations enriched in specific phases of AMD and build transcriptomic signatures of disease, we identified CATCH granularities that isolated high MELD-likelihood scores computed for control, dry, and neovascular AMD conditions. We computed MELD-likelihood scores for each condition on all microglia in AMD (Fig. 3A). Next, we identified a granularity highlighted by topological activity analysis that partitioned regions of high disease likelihood from regions of low disease likelihood (see Methods). With this approach, we identified three clusters, each enriched for a different condition: a cluster enriched for cells from control samples, a cluster enriched for cells from early, dry AMD samples, and a cluster enriched for cells from late-stage, neovascular AMD samples (Fig. 3A).

To identify signatures of AMD present in microglia during the early stage of dry disease pathogenesis, a phase in which microglia have been previously implicated^2^, we performed differential expression analysis between control-enriched and the dry AMD-enriched clusters. Analyzing the top most differentially expressed genes (FDR corrected *p*-value < 0.1) between these subpopulations, a clear activation signature appeared in the early, dry AMD-enriched cluster, including *APOE*, *TYROBP*, and *SPP1* (Fig. 3D), genes known to play a role in neurodegeneration^7^. The association of TYROBP and APOE were validated on sections of human retinal macula by simultaneous immunofluorescence for IBA1, a microglia-associated gene, and in situ hybridization for TYROBP and APOE. On sections of human retinal macula, IBA1-positive cells from patients with dry AMD showed enrichment relative to controls for gene transcripts from *TYROBP* and *APOE*, indicating polarization of a subset of microglia towards the neurodegenerative microglial phenotype in early disease (Fig. 3G). Increased expression of *TYROBP* and *APOE* in microglia was also identified using in situ hybridization on lesions from human brain tissue with early-stage AD and early progressive MS compared with controls (Supplementary Fig. 7C).

Owing to the similarity between this activation state and a previously defined disease-associated microglial state described in mice^7,29^, we performed a comprehensive analysis of microglial states in two other neurodegenerative diseases, AD and progressive MS. Applying the CATCH approach to snRNA-seq data from AD^4^ and MS^5^, we identified all major cell types based on the expression of cell-type-specific marker genes (Supplementary Fig. 6A–D). As in AMD, enrichment analysis revealed that microglia were significantly enriched in AD and MS when compared to control brain tissue (Supplementary Fig. 6E, F). Similar to our analysis of AMD identifying disease-phase-specific transcriptomic states, we applied MELD and topological activity analysis to microglia in the AD and MS datasets and identified three clusters of microglia in each disease: a cluster enriched for cells from control brain tissue; a cluster enriched for cells from early-stage AD tissue or acute active MS lesions; and a cluster enriched for cells from late-stage AD tissue or chronic inactive MS lesions (Fig. 3B, C). Differential expression analysis between the control-enriched and the early-disease-enriched clusters yielded a common shared activation profile in all three diseases when analyzing the top differentially expressed genes (Fig. 3D, middle and right panels) (FDR corrected *p*-value < 0.1)).

To understand the early-disease-enriched microglial populations, we visualized the microglial activation signature (*CD74*, *SPP1*, *VIM*, *FTL*, *B2M*) (*APOE*, *TYROBP*, *CTSB*) (*C1QB* and *C1QC*) as well a homeostatic signature (*P2RY12*, *P2RY13*, and *OLFML3*) on control-enriched and early-disease-enriched clusters from neurodegenerative diseases (Fig. 3E). A clear divergence is seen between the expression pattern of the homeostatic signature in control-enriched populations and early-disease-enriched populations across conditions. With higher expression of activation genes and lower expression of homeostatic genes, the early activated population of microglia display a divergent polarization state. We built a composite microglial activation signature and mapped it onto the clusters along with a previously described disease-associated microglia signature found in an AD mouse model^7^. The early stage of neurodegenerative disease-enriched clusters displayed higher expression of both signatures compared with the control-enriched clusters (Fig. 3F with expression values ranging from 5 to 25 for our activation signature and 7 to 26 for DAM signature).

This shared neurodegenerative microglial phenotype across AMD, MS, and AD involves upregulation of multiple genes implicated in studies of neurodegenerative disease risk. These include *APOE*, a key regulator of the transition between homeostatic and neurotoxic states in microglia^30^ strongly implicated in risk for AD^31,32^ and AMD^33^; *TYROBP* that encodes the TREM2 adaptor protein DAP12, mutations of which are implicated in a frontal lobe syndrome with AD-like pathology^34^ and expression of which is upregulated in white matter microglia in MS lesions; *SPP1* (osteopontin), implicated in microglial activation in brains affected by MS^35^ and AD^36^; and *CTSB*, encoding the major protease in lysosomes cathepsin-B, which is upregulated in microglia responding to *β*-amyloid plaques in AD^36^. Initiation of the pathologic accumulation of extracellular material occurs by different means in these three neurodegenerative diseases. However, the finding that microglial phagocytic, lipid metabolism, and lysosomal activation pathways are upregulated in the early or acute active stage of all three diseases suggests a convergent role for dysregulation in microglia directed towards clearance of extracellular deposits of debris.

### Astrocyte activation signature identified in dry AMD is shared across the early phase of multiple neurodegenerative diseases

**Fig. 4:**
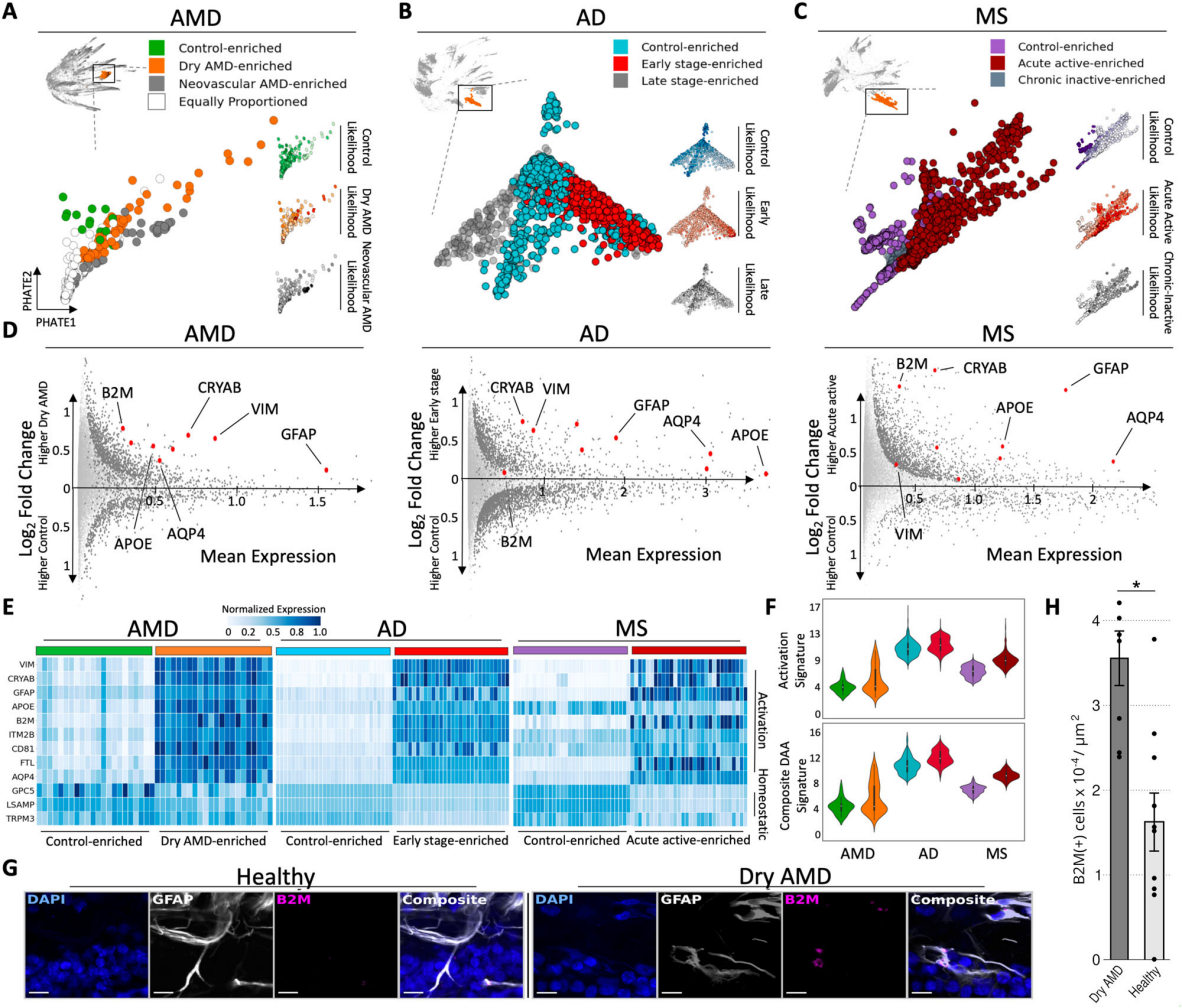
Fine grain analysis of astrocytes reveals a shared activation signature enriched in the early phase of neurodegenerative diseases. **A** 474 astrocytes identified by diffusion condensation at coarse granularity (upper left) can be further subdivided into three clusters at fine granularity, each enriched for cells from a different stage of neurodegenerative disease. Disease state enrichment was calculated using MELD (right) for each condition: Control (top), dry AMD (middle) and neovascular AMD (bottom), with higher MELD likelihoods shown with darker colors. A resolution of the condensation homology, which optimally isolated MELD-likelihood scores from each condition was identified using topological activity analysis. Astrocytes are revisualized using PHATE. **B** As in panel **A**, three subsets of 2361 astrocytes are found in AD with diffusion condensation and topological activity analysis, each enriched for cells from a different stage of AD disease as computed by MELD (right). Astrocytes are revisualized with PHATE. **C** As in panel **A**, three subsets of 5469 astrocytes are found in MS with diffusion condensation and topological activity analysis, each enriched for cells from a different stage of MS as computed by MELD (right). Astrocytes are revisualized with PHATE. **D** Differential expression analysis between control-enriched and early stage of neurodegenerative disease-enriched clusters across neurodegenerative diseases reveals a shared activation pattern in the early stage of disease. This signature includes *B2M*, *CRYAB*, *VIM*, *GFAP*, *AQP4*, *APOE*, *ITM2B*, *CD81*, *FTL*. Significant differentially expressed genes visualized in dark gray (two-sided EMD test with FDR corrected *p*-value < 0.1 as described in methods). **E** Heatmap demonstrating differences in astrocyte expression of the neurodegenerative shared activation pattern and a homeostatic signature between control-enriched and early or acute active disease-enriched astrocytes across neurodegenerative diseases. Color conventions are as in panels A–C. Rows correspond to genes and columns represent individual cells. We have plotted 40 cells from each dataset selected through random sampling to reveal the difference between control-like and early-disease-like cellular states. **F** Composite astrocyte activation signature (top) and disease-associated astrocyte signature (DAA) for the neurodegenerative shared activation pattern in control-enriched cluster and early-disease-enriched cluster across neurodegenerative diseases. Color conventions are as in panels **A**–**C** (*y*-axis—gene expression of signature). Details on statistics are available in methods section. **G** Micrographs of combined in situ RNA hybridization and GFAP immunofluorescence showing more abundant *B2M* expression in astrocyte-rich retinal layers from dry AMD retina when compared to control. All scale bars = 10 μm. **H** Bar plot showing density of *B2M* transcripts in the astrocyte-rich inner plexiform layer, retinal ganglion cell layer, and nerve fiber layers in retina samples affected by dry AMD (*n* = 8 cells) and control (*n* = 10 cells). Data are presented as mean values ± SEM; *p < 1e-03; Welch Two Sample *t*-test.

While astrocyte transcriptomic states and dynamics have been established in mouse models of AD, astrocyte profiles have not been profiled in human AMD lesions at a single-cell resolution^6^. As our initial analysis implicated astrocytes in disease pathogenesis (Fig. 2D, E), we performed similar cross-disease analysis within the astrocyte populations using the CATCH method. Using MELD and topological activity analysis, we identified four clusters of astrocytes at fine granularity within the diffusion condensation hierarchy: a cluster enriched for cells from control samples, a cluster enriched for cells from patients with early, dry AMD, a cluster enriched for cells from patients with late-stage neovascular AMD and a cluster with equal numbers of cells from all three conditions (Fig. 4A). When comparing the transcriptomic profiles of cells within the dry AMD-enriched and control-enriched astrocyte populations, key activation and degeneration-associated genes, such as *GFAP*, *VIM*, and *B2M* were upregulated (Fig. 4D).

Using MELD and topological activity analysis, we identified clusters that isolated stage-specific populations within MS and AD astrocytes. In both diseases, we identified three clusters: a cluster enriched for cells from control brain tissue, a cluster enriched for cells from early-stage AD tissue or acute active MS lesions, and a cluster enriched for cells from late-stage AD tissue or chronic inactive MS lesions (Fig. 4B, C). By comparing the control-enriched and early-disease-enriched clusters within each dataset using condensed transport, we identified a shared gene signature enriched in the early-stage neurodegenerative disease subcluster across all three diseases (Fig. 4E). The integrated gene signature included markers of activated astrocytes, including *VIM*, *GFAP*, *CRYAB*, and *CD81*^37,38^, major histocompatibility complex (MHC) class I (*B2M*)^39,40^, iron metabolism (*FTH1* and *FTL*), a water channel component implicated in debris clearance (*AQP4*)^41^, along with lysosomal activation and lipid and amyloid phagocytosis (*CTSB*, *APOE*). Of interest, many upregulated genes were shared between the microglial and astrocyte early-stage activation signatures, suggesting common glial stress pathways become activated in neurodegeneration.

Similar to microglia, we mapped homeostatic (*GPC5*, *LSAMP*, *TRPM3*) and composite activation signatures (*B2M*, *CRYAB*, *VIM*, *GFAP*, *AQP4*, *APOE*, *ITM2B*, *CD81*, *FTL*) to early-disease-enriched and control-enriched astrocyte clusters across neurodegenerative diseases. Similar to microglia, the composite activation signature and homeostatic signatures were divergently expressed by early enriched clusters (Fig. 4E, F upper with expression values ranging from 0 to 17). Using a recently published disease-associated astrocyte signature established in an AD mouse model^6^, we built a composite activation signature and mapped that onto the early-disease and control-enriched clusters across conditions. The early-disease-enriched clusters displayed higher expression of the disease-associated astrocyte (DAA) gene signature in addition to the composite activation signature (Fig. 4F, lower with expression values ranging from 0 to 16).

To validate the astrocyte signature in tissue, we performed simultaneous GFAP immunofluorescence and RNA in situ hybridization for *B2M*, a component of MHC-I and member of the shared gene signature on sections of the human macula. The retinal layers occupied by GFAP-positive astrocytes (inner plexiform layer to inner limiting membrane) contained a higher density of *B2M* transcripts in retinas affected by dry AMD relative to control retina (*p*-value < 1e-03, two-sided Student’s *t*-test) (Fig. 4G, H).

### Microglia display inflammasome activation signature and astrocytes display pro-angiogenic signature in late-stage neovascular AMD

While glial activation signatures are shared during the early phase of multiple neurodegenerative disease, it is of interest to understand if they persist or evolve in the late-stage of neurodegenerative diseases. To understand these glial activation dynamics across stages of AMD, AD, and MS, we performed differential expression analysis between the early stage of neurodegenerative disease-enriched clusters and the late-stage of neurodegenerative disease-enriched clusters of astrocytes and microglia. Across both comparisons, molecular signatures present in the early stage of AMD, MS, and AD are not detected in microglia and astrocytes during the late-stage of neurodegeneration (Supplementary Fig. 9A, B), indicating transcriptional changes in glia during disease progression.

**Fig. 5:**
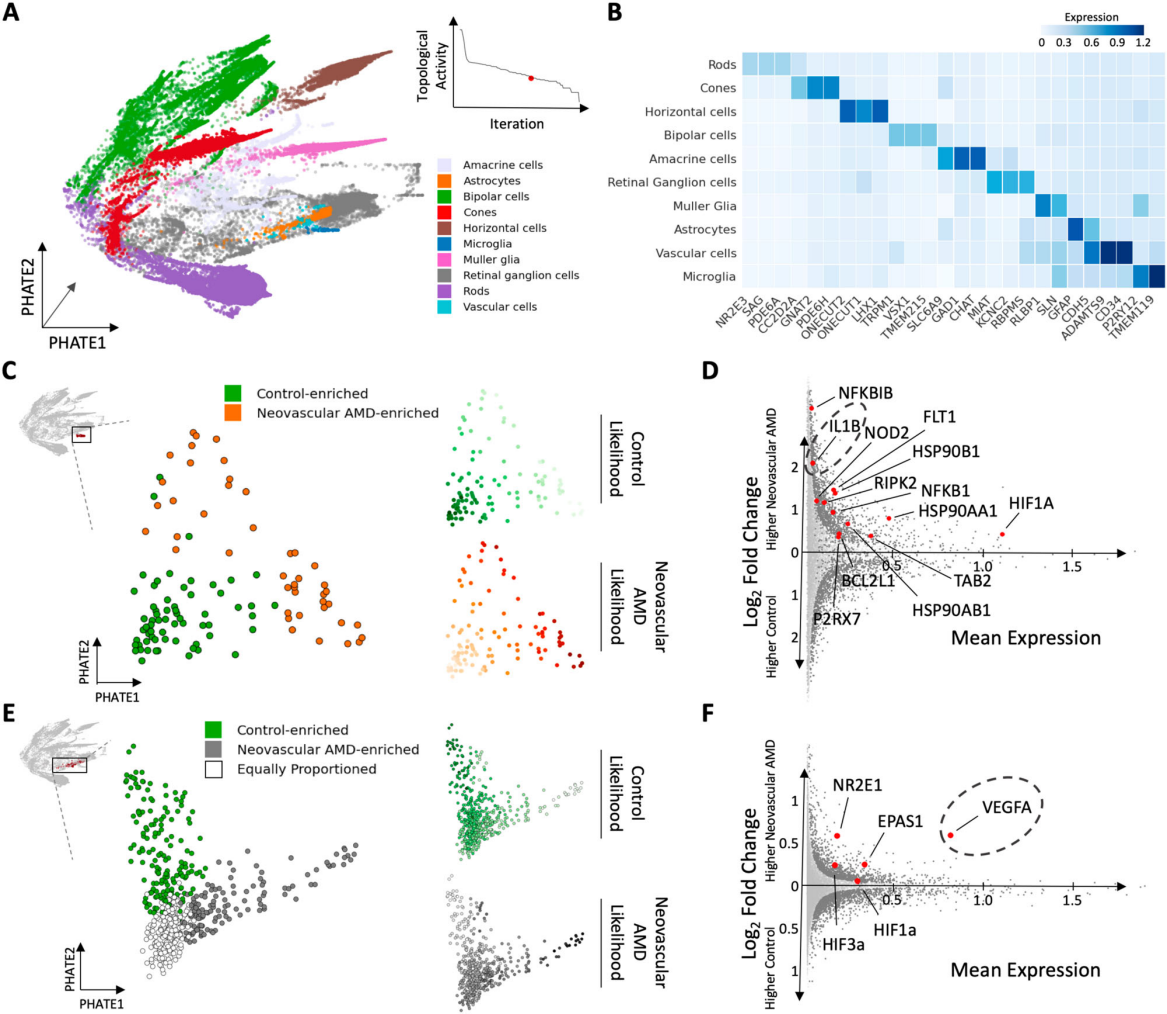
Cell-type-specific changes in gene expression during AMD disease progression. **A** PHATE visualization of 46,783 nuclei isolated from neovascular AMD and control retinas^65^. CATCH analysis identified a resolution of the condensation homology, which isolated cell types. As in Figure 3, each cellular cluster was assigned a cell-type identity based on which gene signature it expressed at the highest level. **B** CATCH-identified cell types, as shown by the average normalized expression of known cell-type-specific marker genes. **C** Disease state enrichment was calculated using MELD (right) for each condition: Control (top), and neovascular AMD (bottom), with higher MELD likelihoods shown with darker colors. A resolution of the condensation homology, which optimally isolated MELD-likelihood scores from each condition was identified using topological activity analysis. Microglia are revisualized using PHATE. Two subsets of microglial cells, one enriched for microglia from retinas with neovascular AMD and another from control retinas. **D** Differential expression analysis between control-enriched and neovascular disease-enriched microglial clusters revealed a different activation pattern in late disease. Significant differentially expressed genes visualized in dark gray (two-sided EMD test with FDR corrected *p*-value < 0.1 as described in methods). This signature includes *NFKBIB*, *IL1B*, *NOD2*, *FLT1*, *HSP90B1*, *RIPK2*, *NFKB1*, *HSP90AA1*, *HIF1A*, *BCL2L1*, *P2RX7*, *TAB2*, *HSP90AB1*. **E** Disease state enrichment was calculated using MELD (right) for each condition: Control (top) and neovascular AMD (bottom) with higher MELD likelihoods shown with darker colors. A resolution of the condensation homology, which optimally isolated MELD-likelihood scores from each condition was identified using topological activity analysis. Astrocytes are revisualized using PHATE. CATCH-identified three subsets of astrocyte cells, one enriched for astrocytes from neovascular retinas, another from control retinas and a third equally split between conditions. **F** Differential expression analysis between the control-enriched and neovascular disease-enriched astrocyte clusters reveals a different activation pattern in late-stage neovascular disease. Significant differentially expressed genes visualized in dark gray (two-sided EMD test with FDR corrected *p*-value < 0.1 as described in Methods section). This signature includes *NR2E1*, *EPAS1*, *VEGFA*, *HIF1A*, *HIF3A*.

To examine the transcriptional changes in glia during progression from early dry to late-stage neovascular AMD pathology, we performed snRNAseq on three additional retinas from human donor retinas with neovascular AMD, and applied the CATCH analysis to 46,783 nuclei when combined with the previously sequenced samples. We identified a granularity of the CATCH hierarchy with low topological activity and assigned cell-type labels based on the expression of cell-type-specific gene signatures (Fig. 5A, B). Following the fine grained CATCH analysis, we identified two clusters of microglia: one cluster enriched for cells from control retinas and one cluster enriched for cells from late-stage, neovascular AMD retinas (Fig. 5C). To identify cell-type-specific transcriptional changes in the subpopulation of microglia enriched in late-stage neovascular AMD pathology, we performed condensation-based differential expression analysis between the control-enriched and the neovascular AMD-enriched clusters. Analysis of the top differentially expressed genes between these subpopulations (FDR corrected *p*-value < 0.1) revealed an inflammasome-related signature including *IL1B*, *NOD2*, and *NFKB1*. The pro-IL-1*β* protein requires both cleavage and release via inflammasome-mediated caspase activation and pyroptosis for bioactivity^42^. Here, activation of inflammasome sensors and oligomerization into proteolytically active complexes may occur in response to a significant and lasting drop in oxygen tension or chronic lipid exposure^42,43^, both known to drive inflammasome activation via NLRP3 (NOD-, LRR- and pyrin domain-containing 3) (Fig. 5D). In late-stage AD and MS alternative cellular stress-associated pathways were upregulated including transcriptional regulators of the ER stress response (*XBP1*) and their target genes involved in protein folding and transport (*HSPA1A*, *HSPA1B*, *HSP90AA1*) and glycosylation (*ST6GAL1* and *ST6GALNAC3*), as well as regulators of autophagy and proteostasis (*ATG7*, *MARCH1*, *USP53*). These signatures highlight a shared cellular stress induction.

Using the fine grained CATCH workflow, we identified two astrocyte subpopulations: one cluster enriched for cells from control retinal samples and one cluster enriched for cells from late-stage, neovascular AMD retinal samples (Fig. 5E). To identify signatures of AMD present in astrocytes during the late-stage of disease pathogenesis, we performed condensation-based differential expression analysis between control-enriched and the neovascular AMD-enriched clusters. Analyzing the top differentially expressed genes (FDR corrected *p*-value < 0.1) between these subpopulations revealed elevation of *VEGFA*, *NR2E1*, and *HIF1A* expression (Fig. 5F), all of which are regulators of cellular responses to low oxygen tension^44–46^. While VEGFA is known to be an important mediator of the abnormal blood vessel growth that characterizes late-stage neovascular AMD and is the target of current therapies for the treatment of disease^33,47,48^, our data demonstrate in humans a specific subpopulation of retinal astrocytes that are a source of this signal.

### Microglia-derived IL-1*β* drives pathologic neovascularization via astrocytes

**Fig. 6:**
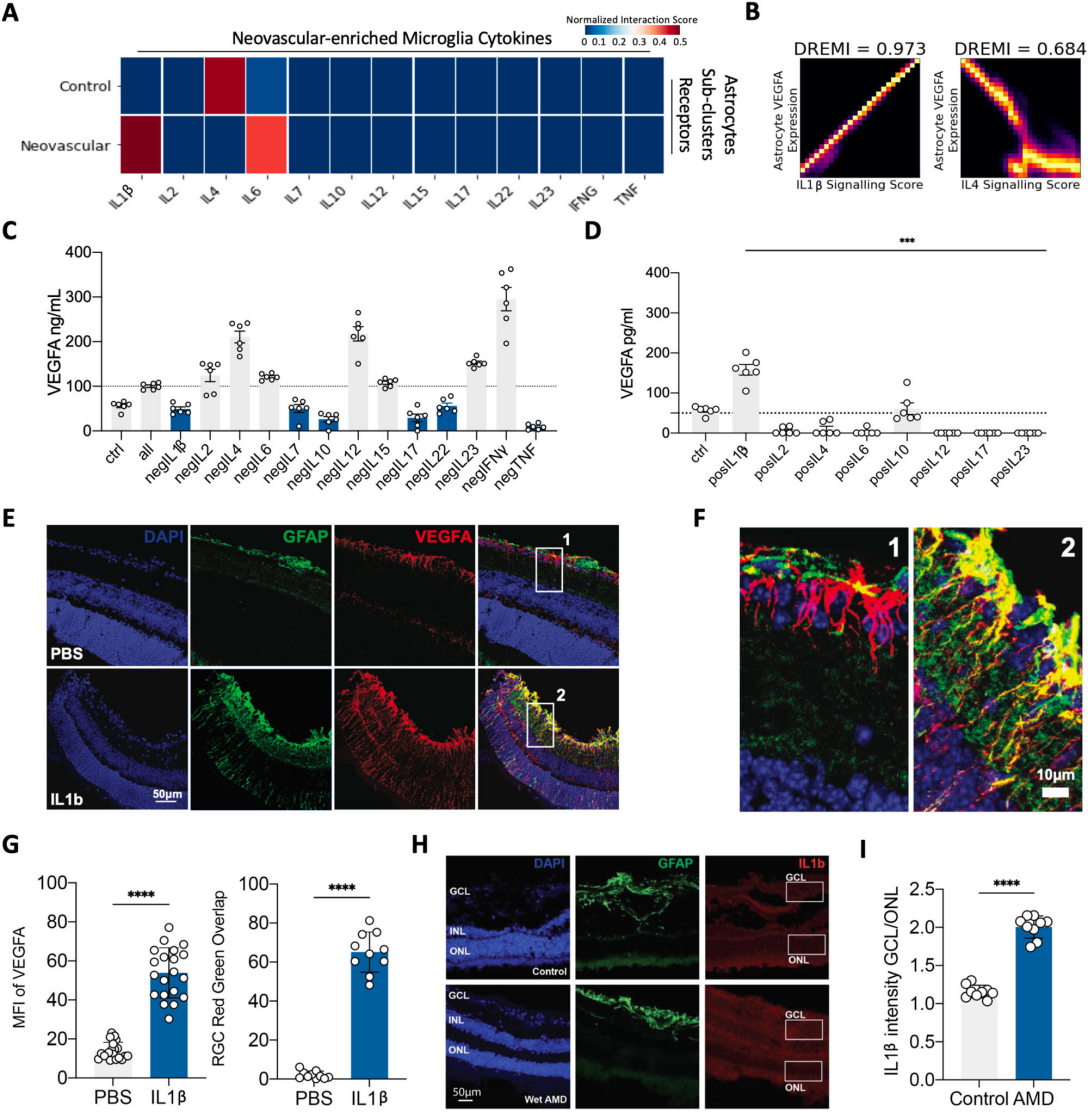
Identifying cytokine regulators of astrocyte VEGFA secretion. **A** Interaction analysis between diffusion condensation identified subtypes of astrocytes and neovascular-enriched microglia (detailed in Fig. 5) computed with CellPhoneDB^52^. Interactions between cytokines produced from neovascular-enriched microglia were computed against cytokine-receptors on astrocyte subtypes. Interactions between specific cytokine-receptor pairs were added to produce a single cytokine interaction value for control and neovascular astrocyte subtypes. **B** DREMI association analysis between astrocyte VEGFA expression, IL-1*β* signaling score, and IL-4 signaling score. Signaling scores for IL-1*β* and IL-4 were computing by adding receptor expression of IL-1*β* and IL-4, respectively, neovascular-enriched astrocytes from Fig. 5. **C** Conducted negative screen in human iPSC-derived astrocytes 24 h after stimulation, subtracting one cytokine (e.g., 'negIL2') from the combinatorial pool to test its necessity in generating a VEGFA-producing astrocyte compared to vehicle control (ctrl). All represents stimulation with a mixture of cytokines (IL-1*β*, IL-2, IL-4, IL-6, IL-7, IL-10, IL-12, IL-15, IL-17, IL-22, IL-23, IFN*γ*, TNF). VEGFA protein is measured using enzyme-linked immunosorbent assay (ELISA). Data were evaluated using one-way ANOVA with multiple comparisons correction using Dunnetts. **D** Conducted single cytokine positive screen in human iPSC-derived astrocytes to test the sufficiency of each cytokine to stimulate astrocyte VEGFA production. VEGFA protein levels are measured using ELISA 24 h after stimulation with each cytokine compared to vehicle control (ctrl). Data were evaluated using one-way ANOVA with multiple comparisons correction using Dunnetts. **E** IL-1*β* or PBS was injected intravitreally into a mouse eye. Retinas were collected 72 h later for immunofluorescent imaging. GCL: ganglion cell layer; IPL: inner plexiform layer; INL: inner nuclear layer; OPL: outer plexiform layer; ONL: outer nuclear layer. PBS phosphate-buffered saline (control). Experiments were repeated at least three independent times with similar results. **F** Zoomed in images of regions indicated in **E**. **G** Quantification of mean fluorescence intensity (MFI) of VEGFA after injection of IL-1*β* or PBS in the mouse eyes after 72 h (left) and quantification of amount of VEGFA and GFAP overlap in the ganglion cell layer of the mouse retina after injection of IL-1*β* or PBS (right). The center of the error bars is the mean. A two-sided Student’s *t*-test was performed. **** represents *p* < 0.0005. **H** Immunofluorescence imaging of human postmortem control and neovascular AMD retinas. Experiments were repeated at least three independent times with similar results. **I** Quantification of IL-1*β* intensity in the ganglion cell layer (GCL) over the outer nuclear layer (ONL) of the retina from **F**. Data are presented as mean values ± SEM; ****p < 0.0005; two-tailed unpaired Student’s *t*-test.

As microglia are known to influence astrocyte functional states through the secretion of soluble factors, we wanted to determine if microglia-derived cytokines could drive VEGFA expression from retinal astrocytes^49–51^. Since CATCH was able to isolate astrocyte and microglial states, we utilized CellPhoneDB interaction analysis^52^ to create a putative list of possible microglia-derived cytokines that may interact with astrocytes to drive VEGFA expression (Fig. 6A). From this analysis, the neovascular-enriched microglia cluster interacted most significantly with astrocytes through IL-1*β* and IL-6, while in controls, microglia-astrocyte interaction was primarily mediated by IL-4. Furthermore, IL-1*β* interacted most significantly with the neovascular-enriched astrocyte subpopulation. Using conditional-Density Resampled Estimate of Mutual Information (DREMI), a method to identify non-linear associations in data^53^, we find that IL-1*β* signaling on astrocytes was most significantly associated with astrocyte production of VEGFA. Meanwhile IL-4 signaling was most significantly associated with a decrease in astrocyte VEGFA production (Fig. 6B). We then set out to validate the cytokine regulators of astrocyte VEGFA production in an unbiased manner.

Cytokines are a part of a complex network of proteins that can produce additive, synergistic, or antagonistic effects. To demonstrate this relationship, we used two screening methods. We first used a combinatorial screening approach utilizing all cytokines identified in our snRNAseq dataset, removing one at a time to test its necessity in creating a VEGFA expressing astrocyte. Screening with human iPSC-derived astrocytes demonstrated that IL-1*β*, IL-10, and IL-17 are positive regulators of VEGFA production in these cells as their subtraction causes decreased VEGFA compared to human iPSC-derived astrocytes stimulated with all cytokines (Fig. 6C). We then tested the sufficiency of some of these cytokines being able to regulate VEGFA production by completing a single protein stimulation and noted that only IL-1*β* caused astrocyte VEGFA secretion (Fig. 6D). Across both analyses, IL-1*β* positively regulated induction of VEGFA from astrocytes both in vitro (Fig. 6C, D) and in silico (Fig. 6B). Our analysis of VEGFA regulation validated the computational prediction of IL-4 being a negative regulator of VEGF-A production (Fig. 6B, C), showing the utility of our approach in identifying signaling interactions between cellular subsets identified with CATCH.

With identification of cytokine mediators of astrocyte VEGFA production, we validated our findings in vivo by injecting IL-1*β* intravitreally in a mouse. This resulted in upregulation of VEGFA (Fig. 6E, F). Not only was there an increase in the amount of VEGFA (Fig. 6G, right), there was an increase of overlapping signals of GFAP and VEGFA, indicative of astrocyte VEGFA activation and secretion (Fig. 6G, left), along with VEGFA expression extending from ganglion cell layer localization down to other layers of the retina. A similar trend was also observed in the adjacent retinal pigment epithelium (RPE), but did not reach statistical significance (Supplementary Fig. 8), likely due to variation in intrinsic autofluorescence among RPE cells. Altogether, this demonstrated the sufficiency of cytokines such as IL-1*β* to induce VEGFA secretion in astrocytes in vitro and in vivo. Cytokines such as IL-1*β* are increased in the vitreous of patients with neovascular AMD^54^, but source and the role of these cytokines in angiogenesis has not been explored. We undertook immunohistochemical staining for IL-1*β* in retinal samples from the macula of patients with AMD and healthy controls, observing that there was an increased amount of IL-1*β* intensity in the inner retinal layers, where astrocytes reside (Fig. 6I). Furthermore, upregulation of VEGFA was seen in these areas (Fig. 6G), indicating that the phenomenon we observe in vitro and in mice likely occurs in human neovascular AMD as well (Fig. 6G–I).

## Discussion

Here, we used snRNA-seq to generate a single-cell transcriptomic atlas of AMD during pathological progression, as well as develop a machine-learning pipeline that allows for meaningful comparisons between cell types and states across diseases and phases. To generate rich signatures for cross-disease comparison among rare cellular subpopulations, we developed a topology-inspired suite of machine-learning tools for single-cell analysis, ‘CATCH’, a tool that identifies cellular subpopulations enriched in a specific condition by computing the complete hierarchy of cellular states using ‘diffusion condensation.’ This pipeline identified cell states enriched in disease, characterized pathogenic expression signatures, and predicted cellular interactions between pathogenic populations, uncovering potential therapeutic targets.

Using CATCH, we identified and characterized specific subpopulations of microglia and astrocytes enriched in the early stage of dry AMD displaying activation signatures related to phagocytosis, lipid metabolism, and lysosomal function. We found similar populations of microglia and astrocytes in analyses of previously published AD and MS single-cell data. While initial inciting events likely differ between neurodegenerative conditions, lipid-rich extracellular plaques play a prominent role in each condition. It is likely that glial cells coordinate clearance of extracellular debris and, in turn, become activated. While the initial phagocytic clearance may be beneficial, glial activation has been shown to play a role in degeneration in AMD, AD, and MS. In later stages of disease, this shared activation landscape evolves. In advanced neovascular AMD, our analysis identified a microglia inflammasome-related signature that drives pro-angiogenic astrocyte polarization and pathologic neovascularization. Microglial inflammasome activation and subsequent IL-1*β* release could be mediated by a variety of signaling sensors. The NLRP3 sensor may be activated in response to a variety of stress signals, including extended lipid exposure or prolonged hypoxia, and has been previously implicated as a microglial driver of neurodegenerative immunopathology, making it a likely candidate^55^. Microglia are highly mobile cells and responsive to a wide variety of stimuli. While lineage tracing that definitively differentiates mononuclear phagocyte origin into circulating macrophages, tissue resident macrophages, and microglia remains challenging, it is believed that the mononuclear phagocytes found at the apical side of the RPE in the vicinity of drusen, which induce activation of the inflammasome, come from all three populations^56^. Furthermore, emerging data suggests that the inflammasome and IL-1*β* have critical roles in promoting degeneration in MS and AD^10–12^. IL-1*β* treatment of RPE cells in vitro results in upregulation of VEGFA expression^57^. Thus, our results implicate this immune sensor in AMD as well.

This set of analyses has clear implications for potential therapeutics for AMD and other neurodegenerative diseases. Currently, anti-VEGF therapy is the primary intervention approved to treat AMD and is only effective in the most advanced stage of disease. Our unbiased analysis not only identified the cell-type specificity of *VEGFA* expression but also identified pathogenic signaling interactions that promote AMD disease progression. Given that VEGFA is a freely diffusible glycoprotein, its production from retinal astrocytes can induce angiogenesis from the choroid. Currently, therapies that inhibit IL-1*β* are available and used in clinical practice for the treatment of other diseases. Inhibiting microglia-derived IL-1*β* in neovascular AMD could provide therapeutic benefit, preventing further neovascularization in advanced patients, or even preventing neovascularization before it begins in patients with earlier stages of disease. Since these mechanisms are shared across MS and AD, it is plausible that these interventions could provide benefit to patients suffering from other neurodegenerative conditions as well. Identifying promising therapeutic candidates to test in neurodegenerative disease clinical trials remains important, and our data suggest that approaches targeting glia may be broadly applicable to multiple neurodegenerative diseases.

## Methods

### Ethics statement

This study, acquisition, and use of postmortem human retinal samples was approved by the Yale Human Research Protection Program’s Institutional Review Board (Yale Protocol Number 2000028616). We complied with all relevant ethical regulations for work with human participants. All human tissue samples were obtained with informed consent prior to tissue collection from participants if enrolled antemortem or legal guardians if postmortem. Mouse experimental protocols were approved by Yale University’s Institutional Animal Care and Use Committee (Yale Protocol Number 2022-20275). All experiments were performed in accordance to the guidelines outlined by Yale University’s Institutional Animal Care and Use Committee.

### CATCH analysis details

The CATCH framework constitutes a group of topologically inspired machine-learning tools to identify, characterize and compare condition-enriched populations of cells across the cellular hierarchy. This framework is centered around the diffusion condensation process, which learns the structure of data across granularities. Beyond making significant adaptions to diffusion condensation, we have introduced tools to help analyze the rich amount of multigranular information produced by diffusion condensation: cellular hierarchy visualization, topological activity analysis, automated cluster characterization and differential expression analysis.

In the following sections, we provide a thorough description of each aspect of CATCH. This includes detailed descriptions of the diffusion condensation process as well as its relationship with MELD, Wasserstein earth mover’s distance (EMD) and topological activity analysis. We complete this section with a rigorous set of comparisons to benchmark our method.

#### Background in manifold learning and diffusion filters

Many of the core concepts in diffusion condensation and its adaptions presented here are based on advances in manifold theory and graph filters. Typically, *n*-dimensional data *X* = {*x*_1_, …, *x*_*N*_} can be modeled as originating from a *d*-dimensional manifold M^*d*^ collected via a non-linear function *x*_*i*_ = *f*(*z*_*i*_). This is because data collection strategies (such as single-cell RNA-sequencing) create high-dimensional observations even when the intrinsic dimensionality is relatively low. Algorithms that use this manifold assumption^58–61^ leverage the intrinsic, low-dimensional geometry of the manifold to explore relationships in data. Diffusion maps^59^ presented a framework that captures intrinsic manifold geometry using random walks that aggregate local relationships between data points to reveal non-linear geometries. These local relationships, known as affinities, are constructed using a Gaussian kernel function:

1 $${{{{{{{\bf{K}}}}}}}}({x}_{i},\, {x}_{j})=\exp \left(-\frac{\parallel {x}_{i}-{x}_{j}{\parallel }^{2}}{\varepsilon }\right)$$

where **K** is an *N* × *N* Gram matrix and bandwidth parameter *ε*, which controls locality. A diffusion operator is defined as the row normalization of the *N* × *N* Gram matrix **K**:

2 $${{{{{{{\bf{P}}}}}}}}={{{{{{{{\bf{D}}}}}}}}}^{-1}{{{{{{{\bf{K}}}}}}}}$$

where **D**(*x*_*i*_, *x*_*i*_) = ∑_*j*_**K**(*x*_*i*_, *x*_*j*_). The diffusion operator matrix **P** represents single-step transition probabilities for a Markovian random walk or diffusion process. Furthermore, as shown in^59^, powers of this diffusion operator **P** (represented as **P**^*t*^ where *t* > 0) represent a *t*-step random walk.

Recent works in data diffusion^27,62–64^ have shown that this framework proposed by^59^ can be used as a low-pass filter when the operator **P** is directly applied to data features, effectively moving data points close to their diffusion neighbors on the manifold. This low-pass filtering process effectively removes high-frequency variation, or noise, and maintains only the principle low-dimensional geometry of the data manifold.

#### Overview of diffusion condensation and its limitations

Diffusion condensation is a dynamic process that builds upon previously established concepts in diffusion filters, diffusion geometry and topological data analysis. The algorithm slowly and iteratively moves points together in a manner that reveals the topology of the underlying geometry. The diffusion condensation approach involves two steps that are iteratively repeated until all points converge:

1. Compute a time inhomogeneous Markov diffusion operator from the data;
2. Apply this operator to the data as a low-pass diffusion filter, moving points towards local centers of gravity.

As established in prior work^8,17,27^, the application of the operator **P** to a vector **v** averages the values of **v** over small neighborhoods in the data. When applied directly to a coordinate function, this application condenses points towards local centers of gravity as determined by bandwidth parameter *ε*, creating a filtered set of coordinates. In this process, if *X*(0) = *X* is the original dataset with diffusion operator **P**_0_ = **P**, then $X(1)=\bar{X}=X(0)*{{{{{{{{\bf{P}}}}}}}}}_{0}$. While previous applications of diffusion filters simply apply one iteration of this diffusion filtering process to data, we can iterate this process to further reduce the variability in the data by computing the Markov matrix **P**_1_ using the coordinate-filtered *X*(1). A new filtered coordinate representation *X*(2) is obtained by applying **P**_1_ to the coordinate functions of *X*(1). Initial applications of the diffusion operator **P** to *X* dampens high-frequency variations in the coordinate function, efficiently moving similar points close to one another. Later applications dampen low-frequency variation, moving similar groups of points towards one another. A more complete explanation of diffusion condensation and its mathematical properties can be found in ref. ^8^ and ref. ^17^.

In its original form, the diffusion condensation process cannot be applied to scRNAseq data. While useful for general data analysis tasks, this process has limitations:

1. the approach does not work in the non-linear space of the single-cell transcriptomic manifold;
2. does not scale to even thousands of data points;
3. does not identify granularities of the topology, which meaningfully partition the cellular state space and
4. does not identify pathogenic populations implicated in disease processes.

In this work, we address each of these limitations and further extend the framework to efficiently perform key single-cell analysis tasks such as cluster characterization and differential expression analysis.

To address these concerns, we have made the following significant adaptions for application to single-cell data:

1. Dynamically learn the geometry of the single-cell manifold with each diffusion filter using *t*-step random walks optimized with spectral entropy;
2. Visualize learned hierarchy via embedding the condensation tree;
3. Use topological activity to identify meaningful granularities for downstream analysis;
4. Implement diffusion operator landmarking, weighted random walks and data merging to efficiently scale to thousands of cells;
5. Implement diffusion condensation with alpha-decay kernel for automated cluster characterization and efficient computation of differentially expression genes.

#### Manifold-intrinsic diffusion condensation learns cellular hierarchy from single-cell transcriptomic data

**Box 1**

##### Algorithm 1

Manifold-intrinsic Diffusion Condensation

**Input:** Cell-by-PC data matrix **X**, initial kernel bandwidth parameter *ε*_0_ and merge threshold *ζ*

**Output:** cluster labels by iteration

1: **X**_0_ ← **X**, *i* ← 0

2: while number of points in **X**_*i*_ > 1

3: Merge data points *a*, *b* if ∣∣**X**_*i*_(*a*) − **X**_*i*_(*b*)∣∣_2_ < *ζ*, where **X**_*i*_(*a*) is the *a*-th row of **X**_*i*_

4: Update the cluster assignment for each original data point based on merging

5: **D**_*i*_ ← compute pairwise distance matrix from **X**_*i*_

6: **K**_*i*_ ← alpha-decay kernel affinity(**D**_*i*_, *ε*_*i*_)

7: **P**_*i*_ ← row normalize **K**_*i*_ to get a Markov transition matrix (*diffusion operator*)

8: **t**_*i*_ ← spectral entropy of **P**_*i*_

9: $${{{{{{{{\bf{X}}}}}}}}}_{i+1}\leftarrow {{{{{{{{\bf{P}}}}}}}}}_{i}^{{{{{{{{{\bf{t}}}}}}}}}_{i}}{{{{{{{{\bf{X}}}}}}}}}_{i}$$

10: *ε*_*i*+1_ ← update(*ε*_*i*_)

11: *i* = *i* + 1

12: end while

Our implementation of diffusion condensation algorithm takes a cell-by-principal component matrix **X** (typically first 50 components) and computes a diffusion operator **P**, representing the probability distribution of transitioning from one cell to another in a single-step using a *α*-decay kernel function with fixed bandwidth *ε* (Alg. 1: Steps 5-7). While other manifold-learning techniques abstract the data to a point where derived manifold-intrinsic features have an unclear relationship with gene expression, our approach learns the manifold while working in principal components, which have a clear relationship with genes. By using the principal components as the substrate for condensation, we can easily characterize clusters and perform differential expression analysis in gene expression space in downstream analysis.

Another key improvement we make in the condensation algorithm is raising **P** to the power of *t* (rather than 1 as in^8^), simulating a *t*-step random walk over the data. This approach adaptively denoises and refines these transition probabilities across iterations such that transitions occur on the non-linear single-cell manifold^27,59,65^. This *t*-step diffusion operator **P**^**t**^ are applied to the input data, acting as a manifold-intrinsic diffusion filter, effectively replacing the coordinates of a point with the weighted average of its *t*-step diffusion neighbors. We track the values of *t* computed across iterations and perform an ablation study to show the necessity of adaptively tuning *t* in each iteration of the manifold-intrinsic diffusion condensation (Supplementary Fig. 2A, B). See Alg. 1 for pseudocode of this algorithm. When the distance between two cells falls below a distance threshold *ζ*, cells are merged together, denoting them as belonging to the same cluster going forward (Alg. 1: Steps 3,4). It is important to note that in the original work,^8^ did not merge points. This process is then repeated iteratively until all cells have collapsed to a single cluster. This merging step, implemented in our manifold-intrinsic diffusion condensation approach, allows for the fast computation of the cellular hierarchy during coarse graining. When applying this manifold-intrinsic diffusion condensation process to single-cell transcriptomic data, we can see cells condense to cluster centroids across iterations, efficiently and rigorously learning the hierarchy of single-cells (Fig. 1C). Finally, through scalable implementation tricks, such as diffusion operator landmarking^66^ and weighted random walks, we have allowed diffusion condensation to scale to thousands of single cells (Supplementary Fig. 2F). Additional details on the selection of *t* as well as scalable implementation tricks can be found below.

#### Learning manifold geometry dynamically with spectral entropy and *t*-step diffusion filters

While the initial implementation of diffusion condensation was created to understand multigranular structure of linear data, single cells occupy a highly non-linear space requiring manifold-learning strategies^27,59,65^. In single-cell data, technical noise, such as drop out and variation, creates measurement artifacts. When building diffusion probabilities on this sort of noisy data, high transition probabilities can be calculated between unrelated cells inappropriately. Thus, directly working with **P**, fails to acknowledge non-linearities and technical artifacts present within single-cell data. Previous work in data diffusion has shown that raising the diffusion operator **P** to the power of *t* refines these transition probabilities, increasing the chance of transitioning to more related cells^27,59,65^. This powering step allows learning of the relevant non-linear geometry of the data manifold, allowing us to ignore spurious neighbors found in the ambient measurement space of cells and instead finding diffusion neighbors that lie on the single-cell manifold.

As single-cell datasets can often suffer from different types and scales of noise, previous approaches have found that the correct number of *t*-steps to take must be computed adaptively in a data dependent manner^27,67^. Previously proposed strategies to select *t* however, are often slow, as they require trial-and-error approach, which rely upon the structure of the underlying dataset. In diffusion condensation, however, the structure of the underlying dataset continuously shifts between granularities due to the repeated application of diffusion filters, making the repeated computation of *t* necessary and through these techniques computationally unwieldy. Therefore, we propose to select *t* adaptively at each condensation iteration by using a *spectral entropy*-based approach. Previously, it has been shown that powering the diffusion operator **P** differentially effects the eigenvectors of the powered matrix. While the noisy, high-frequency eigenvectors rapidly reduce to zero, the more informative, low-frequency eigenvectors diminish much less rapidly^27^. We reason that there is a value of *t*, which optimally reduces the noisy information from the high-frequency eigenvectors while maintaining the maximum information from the low frequency, informative eigenvectors. To identify this point, we compute the spectral entropy of the diffusion probabilities **P** when powered to different levels of *t*.

Spectral entropy is defined as the Shannon entropy of normalized eigenvalues, i.e.,

3 $$S({{{{{{{\bf{P}}}}}}}},\, t)=-\mathop{\sum}\limits_{i}{\psi }_{i}^{t}\log ({\psi }_{i}^{t}),$$

As there is a degree of information loss with each increasing value of *t*, we try to identify the point at which this information loss curve stabilizes. While powering to low values of *t* rapidly decreases spectral entropy as large amount of noise diminish, powering to higher values of *t* only slowly reduces entropy due to the slower removal of information from informative, low-frequency eigenvectors. Taking the point at which this stabilization occurs as done in ref. ^65^, optimally allows us to adaptively select a value of *t* at each diffusion condensation iteration, allowing us to produce a diffusion filter, which has learned the single-cell manifold.

In fact, deriving *t* adaptively in a data-driven manner is critical to learning the multigranular cluster structure of data. In order to illustrate this point, we generated synthetic single-cell data using Splatter^19^. As can be seen, across differing amounts of variational and drop out noise, optimally selecting *t* via spectral entropy produces a better set of cluster labels than when setting *t* in a fixed, user-determined manner (Supplementary Fig. 3B). In fact, we can see that setting *t* to 1 does not learn the data manifold or the cluster structure of even fairly noiseless single-cell data, revealing the need for selecting a high level of *t* in an adaptable, data-driven manner. Finally, we see that over successive condensation steps, the complexity of the data decreases and thus requires lower levels of *t* to learn (Supplementary Fig. 3A).

#### Improving scalability with weighted random walks, landmarked diffusion operators and merged data points

Repeated computation of a diffusion operator from high-dimensional single-cell data, powering of this diffusion operator to identify the optimal value of *t* followed by diffusion filter application via matrix multiplication is computationally expensive. Repeating these computations, potentially hundreds of times, as done by diffusion condensation is unwieldy. In fact, this approach, in its most basic implementation, scales very poorly to high-dimensional single-cell data with tens of thousands of features and potentially hundreds of thousands of cells. To improve computational efficiency, we perform the following steps:

1. Merge points together that fall below a preset distance threshold *ζ* to create a cluster and weighting random walks to maintain effect of data density;
2. Compute compressed diffusion operator through landmarking^66^ to efficiently compute spectral entropy as done in ref. ^65^.

Collectively, these advances drastically improve the computational speed of diffusion condensation (Supplementary Fig. 2F). In practice, a complete cellular hierarchy of a 13,000 cell dataset can be analyzed within 6 min in a Google Colaboratory notebook (a service which provides 4-core 2-GHz CPU and 20 GB of RAM for free).

#### Visualizing and analyzing condensation tree with topological activity analysis to identify meaningful granularities for downstream analysis

Topological data analysis (TDA) is a powerful framework that learns and analyzes data across granularities. In TDA, one identifies related data points by identifying all pairs whose distance falls below a distance threshold *δ* in a distance matrix **D**. Any pair of points that falls below this threshold is deemed to be part of the same *connected component* or cluster. As *δ* increases, more cell pairs will be connected, quickly creating fewer connected components, or fewer larger clusters, at coarser granularities. In topological data analysis, *persistent homology* is a principled approach to track the connected components that are created and destroyed across a range of granularities. While diffusion condensation learns the multigranular structure of data through a cascade of non-linear diffusion filtration approach instead of an increasing distance threshold, these approaches are intuitively related.

We can study this diffusion condensation process either in a holistic manner, evaluating all granularities simultaneously, or in a detailed manner, by evaluating meaningful granularities independently. At a high level, the cellular hierarchy can be studied by visualizing the cellular hierarchy, containing all merges across all granularities. As manifold-intrinsic diffusion condensation operates in PCA dimensions, we practically implement this visualization by stacking the first two axes of **X**_*i*_ → **X**_*i*+1_ ⋯ **X**_*I*_, creating a hierarchical tree that summarizes the cluster structure of the data across granularities (Fig. 1D-i).

For more detailed analysis, we can cut this hierarchical tree at meaningful levels to identify granularities of clusters that optimally partition cells into meaningful clusters based on the data geometry. Using *persistent homology*, we define a *topological activity analysis*, a technique to analyze the creation and destruction of clusters across consecutive iterations (**X**_*i*_ → **X**_*i*+1_) of the manifold-intrinsic diffusion condensation process. Topological activity analysis is a variation of the total persistence summary statistic often used to characterize topological activity in classical topological data analysis^68^. In this analysis framework, we summarize the merging of points during the condensation process and assign each cluster a topological ‘prominence’ value known as *persistence*. Highly persistent components are taken to represent groups of cells that are similar in their transcriptional profile and distinct from other cells. These clusters, and their associated persistence values, are best represented using a ‘persistence barcode.’ This is a visualization^69^ consisting of horizontal bars of different lengths; each bar corresponds to one topological feature—a subgroup of cells in our case—while the length of each bar depicts the persistence of that feature, directly indicating to what extent the feature is prominent. Assuming that the persistence barcode consists of a set of bars with end coordinates ${{{{{{{\mathcal{B}}}}}}}}:=\{{b}_{1},\ldots,{b}_{k}\}$, we calculate an activity curve **A**: R → N defined by ${{{{{{{\bf{A}}}}}}}}(i):=|\{b\in {{{{{{{\mathcal{B}}}}}}}}|b\le i\}|$, i.e., the number of topological features (cell clusters) that are active and independent at a given iteration *i*. This activity curve, first proposed by^70^ and implemented by^71^, allows us to identify iterations of rapid condensation as well as iterations of relative inactivity through the gradient of **A**. Specifically, we are interested in contiguous segments in the preimage of ∂**A**/∂*i* = 0, which we refer to as *i*-segments. The length of an *i*-segment is the number of iterations for which there is no change in topological activity. Thus, the number of iterations for which ∂**A**/∂*i* = 0 provides a principled way of selecting meaningful condensation granularities computed by the diffusion condensation process. Inspired by the nomenclature of persistent homology, we refer to the length of a *i*-segment of no topological activity as its persistence, meaning that we are looking for the most persistent of such topological activity segments.

#### Identification of disease-enriched populations in conjunction with MELD

While analysis of the cellular hierarchy will identify populations of related cells in an unbiased and multigranular manner, it does not use condition of origin information to identify cellular populations that are enriched in disease conditions of interest. While we can integrate cells from different disease conditions in our analysis, cells of a certain pathogenic transcriptomic state may be over represented in a submanifold of a given cell type. By comparing the cells of a particular type directly to each other based on condition of origin, we dilute out this enrichment information and lose important signal. In fact, identifying these pathogenic states and comparing them directly with clustering and differential expression tools has been shown to be a more powerful method to identifying condition-enriched cell states and expression signatures^9,72^. We explore this point later in this section.

To take condition-specific information into consideration, we use MELD to identify cellular populations that are enriched or depleted in different disease phases^9^. MELD is a manifold-geometry-based method of computing a likelihood score for each cell, indicating whether it is more likely to be seen in the normal or diseased sample. Finding a clustering method that separates these condition-enriched groups is a difficult problem that needs to be performed to identify discrete cellular populations, which can be thoroughly described. To rigorously identify cell populations with strong disease-specific enrichment signals, we combine this cell-level MELD score with information from our topological activity analysis to identify resolutions that produce stable clusters. Then within this stable clustering, we identify populations that are enriched in differing disease conditions.

#### Automated cluster characterization via manifold-intrinsic diffusion condensation

While identification of pathogenic cellular states is critical, biologists are more interested in what defines these populations. Most manifold-learning methods visualize or cluster populations of interest, requiring further expensive computation to characterize cell populations and discover differentially expressed genes. As our approach continuously condenses the transcriptomic profiles of single cells to local cluster centroids in manifold space, at any iteration, the transcriptomic states of the condensed data can be extracted at no additional computational cost. To enhance this convergence to centroids we implement our diffusion condensation process with an *α*-decay kernel (Supplementary Fig. 2C). This kernel more strongly thresholds the conversion of distances to affinities, closely resembling the box kernel, which accurately computes cluster centroids over the course of main point merges. When diffusion condensation merges two cells together at a particular iteration, the newly formed point lies close to the centroid of the original two cells in transcriptomic space. Under specific conditions, the new point is exactly the cluster centroid as delineated in the Proposition below. First, we define the *α*-decay kernel as:

4 $${{{{{{{{\bf{K}}}}}}}}}_{\alpha }({x}_{i},\,{x}_{j})=\exp \left(-\frac{\parallel {x}_{i}-{x}_{j}{\parallel }^{\alpha }}{{\varepsilon }^{\alpha }}\right)\,,\quad i,\,j=1,\ldots,N.$$

The standard Gaussian kernel function as shown in equation (1) has an *α* of 2. The default *α*-decay kernel meanwhile uses a much higher value (default in our implementation is 40), which converts close distances into affinities much more stringently (Supplementary Fig. 2C). As *α* increases to infinity, this kernel function converges almost completely to the box kernel. With this kernel, we are ready to state a set of conditions under which the diffusion condensation process can be easily characterized.

##### Proposition 1

Assume there exists a unique global minimum non-zero distance *δ*_*i*_ between points *x*_*a*_, *x*_*b*_ at each iteration *i*, with the next pair of points at distance at least *δ*_*i*_ + *τ*_*i*_ with 0 < *τ*_*i*_. Note that *x*_*a*_, *x*_*b*_ could have multiplicity greater than 1, representing clusters of size > 1. Then set the bandwidth to *ϵ*_*i*_: = *δ*_*i*_ + *τ*_*i*_/2 at each iteration of the condensation process. For a large enough *α*, the diffusion condensation process will maintain two invariants for the first *N* − 1 steps:

1. The number of points will be *N* − *i*;
2. Unique points will be located at the centroid of their cluster.

##### Proof

It is easy to verify (1) and (2) hold for step zero. For all *i* < *N* and for sufficiently large *α*, ***K***_*α*_(*x*_*k*_, *x*_*j*_) becomes arbitrarily close to 1 for (*k*, *j*) ∈ {(*a*, *a*), (*a*, *b*), (*b*, *a*), (*b*, *b*)} and 0 otherwise. Exactly one merge occurs at each timestep between points at *x*_*a*_ and *x*_*b*_. Given ***P***_*i*_ as described above, they merge to the point $\frac{|{x}_{a}|{x}_{a}+|{x}_{b}|{x}_{b}}{|{x}_{a} |+|{x}_{b}|}$, i.e., the cluster centroid. By induction (1) and (2) hold for all *i* < *N*.  □

In this setting, the condensation process always converges in exactly *N* − 1 steps. In practice, we aim for much shorter convergence times as there are many fewer than *N* − 1 interesting levels of clustering. For 50,498 cells, we find a set of parameters that allow for convergence in 150 steps. For this reason we use a larger bandwidth *ϵ*_*i*_, which leads to much faster convergence and gives cluster centers at each level that are close to but not exactly the cluster centroids of the points they represent. Another factor is the setting of the *α* parameter. Since, manifold-intrinsic diffusion condensation operates in PC dimensions, the complete gene expression profile of cluster centroid *x*_*ab*_ can easily extracted by inverting the PC dimensions. We show that this point is not only mathematically true but also empirically true in practice (Supplementary Fig. 3C).

#### Differential expression analysis via approximation of gene Wasserstein distance

Beyond cluster characterization, differential expression analysis is a critical method to identify signatures of pathogenic populations. Earth Mover’s Distance (EMD), also known as ‘optimal transport’, typically manifested in 1D-Wasserstein distance, is a popular and established method to extract differentially expressed genes between clusters^27,73–75^. EMD, however, is computationally expensive, as it computes an optimal mapping between points, running in $\tilde{O}({n}^{3})$ time. Previously, tree-based implementations like FlowTree^76^ and QuadTree^77^ have been able to closely approximate ground truth Wasserstein distance while significantly improving runtime by constraining the transport of points through the branches of a hierarchical tree^78^. Since diffusion condensation too produces a tree embedding of the data, we utilize tree-based transport for differential expression.

EMD, or 1-D Wasserstein distance, is a measure of distance between two distributions. For a given ground distance, the Wasserstein distance between distributions can be thought of as the minimal total distance needed to move one distribution to the other. Let *μ*, *ν* be two distributions on a measurable space Ω with metric *d*( ⋅ , ⋅ ), and Π(*μ*, *ν*) be the set of joint distributions *π* on the space Ω × Ω, such that for any subset *ω* ⊂ Ω, *π*(*ω* × Ω) = *μ*(*ω*) and *π*(Ω × *ω*) = *ν*(*ω*). The 1-Wasserstein distance *W*_*d*_ also known as the earth mover’s distance (EMD) is defined as:

5 $${W}_{d}(\mu,\,\nu ):\!=\mathop{\inf }\limits_{\pi \in {{\Pi }}(\mu,\, \nu )}{\int}_{{{\Omega }}\times {{\Omega }}}d(x,\,y)\pi (dx,\,dy).$$

When *μ*, *ν* are discrete distributions over points in ${{\mathbb{R}}}^{d}$, of size *m*, *n*, respectively, this can be equivalently expressed in matrix notation as:

6 $$\begin{array}{ll}{W}_{d}(\mu,\,\nu ):=\mathop{\min }\limits_{{{\Pi }}\ge 0}\mathop{\sum }\limits_{i=1}^{m}\mathop{\sum }\limits_{j=1}^{n}{{{\Pi }}}_{ij}d({x}_{i},\, {x}_{j})\\ \mathop{\sum }\limits_{i=1}^{m}{{{\Pi }}}_{ij}={\nu }_{j},\quad \forall j\in \{1,\ldots,n\}\\ {{\mbox{subject to:}}}\mathop{\sum }\limits_{j=1}^{n}{{{\Pi }}}_{ij}={\mu }_{j},\quad \forall i\in \{1,\ldots,m\}\end{array}$$

For general ground distances this is computable using the Hungarian algorithm in $\tilde{O}({n}^{3})$ time. Intuitively, the difficulty in computing the optimal transport is finding the map Π, which optimizes the cost within the constraints. However, for a tree metric, this optimal map is easy to compute in closed form because there is only a single path (through the tree) between pairs of points. This single path between pairs of points results in a reduced computational complexity of $\tilde{O}(n)$. This is best understood using the Kantorovich-Rubinstein dual form of the Wasserstein distance:

7 $${W}_{d}(\mu,\, \nu )=\mathop{\sup }\limits_{f:\parallel f{\parallel }_{L}\le 1}{\int}_{{{\Omega }}}f(x)d\mu -{\int}_{{{\Omega }}}f(x)d\nu$$

where the witness function $f:{{\Omega }}\to {\mathbb{R}}$ and ∥ ⋅ ∥_*L*_ denotes the Lipschitz norm. This dual form holds under a few minor conditions, which hold for the spaces considered here. For more information see^79^.

Given some rooted tree *T* with strictly non-negative edge lengths, we define the natural tree metric *d*_*T*_(*x*, *y*) as the length of the unique path between nodes *x*, *y*. We denote the mass of a distribution on a subtree *T*_*r*_ rooted at node *r* as $\mu ({T}_{r})={\sum }_{x\in {T}_{r}}\mu (x)$. For each node *v* ∈ *T* we denote its associated parent edge as *e*_*v*_ with weight *w*_*v*_. In this setting, it is easy to construct the optimal witness function in eq. (7). Without loss of generality, one starts at the root *r* and builds *f* such that *f*(*r*) = 0 and for each edge *e*(*u*, *v*) where *u* is a parent of *v*, *f*(*v*) = *f*(*u*) + *w*_*e*_ ⋅ sign(*μ*(*T*_*v*_) − *ν*(*T*_*v*_)). Given this construction, it is easy to see that the Wasserstein distance with tree ground distance has the following closed form:

8 $${W}_{{d}_{T}}(\mu,\, \nu )=\mathop{\sum}\limits_{v\in T}{w}_{v}|\mu ({T}_{v})-\nu ({T}_{v})|.$$

The question then comes to: what are useful tree metrics? An ideal tree metric that has low distortion of Euclidean space and is scalable to high dimensions. QuadTree^77^ is a tree metric algorithm designed to approximate the optimal transport distance between discrete measures with Euclidean ground distance by recursively partitioning space into hypercubes, but does not scale well with dimension. Specifically, assume, without loss of generality, that the data lies in the [0, 1]^*d*^ hypercube, then at each level *h* ∈ [0, *H*) divide the space into ${2}^{{d}^{h}}$ hypercubes with side length 2^−*h*^. This forms an H-level tree with each node representing a hypercube.

If the center of the hypercube is randomly shifted, then the QuadTree distance ${W}_{{d}_{QT}}$ has distortion at most $O(d\log 1/\tau )$ where *τ* is the minimum distance between data points, i.e.

9 $$c\cdot (d\log \tau ){W}_{{d}_{QT}}(\mu,\nu )\le {W}_{\parallel \cdot {\parallel }_{2}}(\mu,\nu )\le C\cdot (d\log \tau ){W}_{{d}_{QT}}(\mu,\nu )$$

for some constants *c*, *C* in expectation^77^.

However, QuadTree distance scales poorly as it is computed in $O(Nd\cdot \log (d1/\tau ))$. In the high-dimensional setting, such as snRNAseq data, the poor scaling with respect to *d* both computationally and in the approximation is undesirable. In this setting^78^ suggests sampling trees using furthest point clustering^80^. Furthermore,^76^ implements FlowTree, a small modification to QuadTree that makes tree Wasserstein distances significantly more accurate with the addition of small additional computational cost.

Drawing from both FlowTree and QuadTree, CATCH implements a new formulation of EMD over the diffusion condensation tree. For two diffusion condensation clusters *a*, *b* located at *C*_*a*_, *C*_*b*_, respectively, we define the *condensation-based Wasserstein approximation distance* between them as:

10 $${W}_{CT}(a,\,b,T)=\parallel {C}_{a}-{C}_{b}{\parallel }_{2}+\mathop{\sum}\limits_{e(u,v)\in {T}_{a}}{w}_{e}\cdot a({T}_{v})+\mathop{\sum}\limits_{e(u,v)\in {T}_{b}}{w}_{e}\cdot b({T}_{v})$$

where *w*_*e*_ : = 2^−*h*^∥*C*_*v*_ − *C*_*u*_∥_2_ for edge *e*(*u*, *v*) at depth *h* and *a*(*x*), *b*(*x*) are defined as indicator functions of their respective clusters.

This leads to the following proposition stating that no matter how close we are to the settings in Proposition 1, *W*_*CT*_ still represents a valid tree Wasserstein distance between clusters.

##### Proposition 2

The condensation-based Wasserstein distance approximation distance *W*_*CT*_, for any diffusion condensation tree *T*, defines a valid Wasserstein distance over a tree ground distance for any two clusters in that tree.

##### Proof

We show this by constructing the associated tree metric *d*_*CT*_ on an arbitrary condensation tree *T*_*CT*_ and conclude by showing that ${W}_{{d}_{{T}_{CT}}}$ is equivalent to *W*_*CT*_. Begin by rooting the tree at a node representing *C*_*a*_ with two children, the root of *T*_*a*_ named *r*_*a*_ and *C*_*b*_. The edge *e*(*C*_*a*_, *r*_*a*_) has weight 0 and the edge (*C*_*a*_, *C*_*b*_) has weight ∥*C*_*a*_ − *C*_*b*_∥_2_. The node *C*_*b*_ will have a single child node the root of *T*_*a*_ named *r*_*b*_, and is connected by an edge of length zero. All other nodes will be defined as in *T*_*a*_ and *T*_*b*_ with associated edge weights.

It is easy to verify that the path measure over *T*_*CT*_ construction represents a valid distance *d*_*CT*_. Finally, we verify that the Wasserstein distance with a ground distance of *d*_*CT*_ is equivalent to *W*_*CT*_ as defined in eq. (10). Indeed, because we added a skip connection in the tree to directly connect nodes *a*, *b* with an edge of length ∥*C*_*a*_ − *C*_*b*_∥_2_ and since *a*(*T*_*v*_) for *v* ∈ *T*_*b*_ is always zero and vice versa, we have

11 $${W}_{{d}_{CT}}(a,\,b)	=\mathop{\sum}\limits_{e(u,v)\in {T}_{CT}}{w}_{e}|a({T}_{v})-b({T}_{v})|\\ 	={w}_{e({C}_{a},{C}_{b})}|a({T}_{{C}_{b}})-b({T}_{{C}_{b}}) |+\mathop{\sum}\limits_{e(u,v)\in {T}_{a}}{w}_{e}|a({T}_{v})-b({T}_{v}) |+\mathop{\sum}\limits_{e(u,v)\in {T}_{b}}{w}_{e}|a({T}_{v})-b({T}_{v})|\\ 	=\parallel {C}_{a}-{C}_{b}{\parallel }_{2}|0-1 |+\mathop{\sum}\limits_{e(u,v)\in {T}_{a}}{w}_{e}|a({T}_{v})-0 |+\mathop{\sum}\limits_{e(u,v)\in {T}_{b}}{w}_{e}|0-b({T}_{v})|\\ 	=\parallel {C}_{a}-{C}_{b}{\parallel }_{2}+\mathop{\sum}\limits_{e(u,v)\in {T}_{a}}{w}_{e}\cdot a({T}_{v})+\mathop{\sum}\limits_{e(u,v)\in {T}_{b}}{w}_{e}\cdot b({T}_{v})\\ 	={W}_{CT}(a,b,T).$$

Note that *W*_*CT*_ does not calculate the Wasserstein distance over the same tree for each set of clusters, and as shown in^76^ this often improves the accuracy as compared. In addition, it is useful conceptually but not essential that the cluster centers *C*_*a*_, *C*_*b*_ are near the cluster centroids. In Proposition 1 we delineated the setting where this holds exactly, but these parameters are impractical for our efficient computation requiring *n* − 1 diffusion steps. Instead, we are satisfied with centers that are close to the centroids but are efficiently computable in many fewer diffusion steps. Our formulation is similar to the standard Wasserstein distance with tree ground distance as in eq. (8), but simplified and optimized for the case of comparing clusters, which are elements of the tree metric. We make two changes. First, we add a skip connection in the tree to directly connect nodes *a*, *b* with an edge of length ∥*C*_*a*_ − *C*_*b*_∥_2_ as in ref. ^76^, which is empirically more faithful in their experiments and ours. Next, we note that *a*(*T*_*v*_) for *v* ∈ *T*_*b*_ is always zero and vice versa, thus simplifying the second and third terms. These two optimizations give us an algorithm that is efficient in high dimensions and is effective empirically (Supplementary Fig. 1E and (Supplementary Fig. 2D) across granularities (Supplementary Fig. 2E).

Using this intuition, CATCH is able to rapidly perform differential expression analysis by approximating the Wasserstein metric on a per-gene basis along the hierarchies generated by manifold-intrinsic diffusion condensation. Leveraging our approach’s ability to summarize transcriptomic landscapes with the *α*-decay kernel, we use multiple granularities of the cellular hierarchy to accurately approximate ground truth Wasserstein distance between genes and identify cluster-specific expression signatures^78^ (Fig. 1D-iv). We show that this is empirically true with our comparisons (Supplementary Fig. 2D and Supplementary Fig. 1F).

Inspired by previous statistically sound methods of identifying differentially expressed genes, we implement a resampling-based approach to identify true differentially expressed genes^73,81^. In this approach, we estimate false-discovery rate (FDR), which is the expected proportion of rejected null hypotheses falsely for each gene’s test statistic at a given significance level^73,81^. To calculate FDRs from our Wasserstein values, we generate a null distribution by permuting the cluster labels (in practice 1000 times) and compute Wasserstein distance between the permuted classes each time. Using the median of permuted Wasserstein distances for each gene, we create a null distribution from which we can compute *p*-values per-gene. The attained *p*-values are corrected using the Benjamini–Hochberg procedure^82^.

#### Automated cluster characterization and Earth Mover’s Distance between genes in synthetic and real single-cell data

While manifold-intrinsic diffusion condensation implemented with an *α*-decay kernel can theoretically approximate ground truth cluster characterizations and compute differentially expressed genes, we wanted to demonstrate this reasoning in synthetic and real single-cell data. To empirically show that out condensation-based approach approximates EMD between two clusters, we compute EMD values between genes using Wasserstein optimal transport as well as out approximate approach on synthetic and real data using Gaussian and *α*-decay kernel implementations of diffusion condensation. Using single-cell data generated from splatter, we compute diffusion condensation and identified the granularity with the highest topological persistence using topological activity analysis. We then computed ground truth and approximate differential expression values by comparing every cluster at this granularity with every other cluster. In our analysis, a total of 12,130,200 and 4,535,640 gene comparisons were computed using Gaussian and *α*-decay approaches, respectively. Comparing both Gaussian and *α*-decay approximate Wasserstein distances against ground truth per-gene Wasserstein values, we can see the value in our *α*-decay approach (Supplementary Fig. 2D) as it approximates ground truth Wasserstein distance with a correlation coefficient of 0.979. Furthermore, our approach computed all 4,535,640 gene comparison in 63 s while ground truth values were computed in 43,125 s, equating to a 684 fold increase in computational speed.

We repeated our comparison in real single-cell data, again comparing both approaches to ground truth Wasserstein EMD values, this time across 10 granularities identified by topological activity analysis. As previously performed, at each granularity, all clusters were compared to all other clusters using each approach. Across all comparisons, a total of 10,166,640 and 2,541,660 comparisons were computed for the Gaussian and *α*-decay implementations, respectively. Again we see that *α*-decay is critical to accurately capturing ground truth EMD values, with our *α*-decay approach correlating highly with ground truth EMD while Gaussian approach was less correlated (Supplementary Fig. 1F). Furthermore, we again see an increase in computational speed with our condensation-based approach. In our weighted implementation, we are able to compute all 2,541,660 comparisons in 32 s, while ground truth EMD values were computed in 27,517 s, equating to a similar 860 fold increase in computational speed. Next, we show that this correlation between ground truth EMD and condensation-based Wasserstein distance approximation is not a feature of cluster granularity as defined by number of cluster (Supplementary Fig. 3D). Finally, we also use *α*-decay and Gaussian implementations to compute and compare cluster characterizations to ground truth in real single-cell data. Using the same set of clusters and granularities as previously computed, we see that *α*-decay kernel again more accurately characterizes clusters than a Gaussian kernel (Supplementary Fig. 3C).

#### CATCH identifies differentially expressed genes from noisy single-cell data

Previously, disease signatures within a cell type have been determined by comparing cells’ gene expression profiles based on their condition of origin. For instance, microglia would be separated into two groups based on condition of origin, either disease or healthy, which would then be compared. We believe that CATCH improves on this framework by first identifying disease-enriched states and then identifying differentially expressed genes between these states. This is because our procedure accounts for significant noise that can appear in single-cell data to more purely identify cell states enriched in particular disease settings. In fact, previous studies have validated that this approach identifies biological processes better than previous ‘condition-of-origin’ comparison approaches^9^.

To illustrate this point in real single-cell data, we performed differential expression analysis between microglia based on their condition of origin across all three neurodegenerative disease datasets. We reason that if our approach is more sensitive to identify differentially expressed genes, a less sensitive approach would not find as strong of a shared signature. After setting significance cutoffs based on our per-gene false-discovery rates, we identified significantly enriched genes in the early or acute active phase of each disease (Supplementary Fig. 10a). However, across all comparisons, we identified significantly fewer differentially expressed genes in this cell-type analysis (135, 68, and 416) than with our pipeline (618, 795, and 1551 for AMD, AD, and MS, respectively), indicating that the identification of pathogenic cellular subtypes with CATCH before comparison increases our ability to detect differentially expressed genes. In cross-disease comparisons among early-stage neurodegenerative microglia, only 17 common genes were found, significantly less than the 168 common genes found with our pipeline. Of the common genes, only half of the activation signature was found (*APOE*, *B2M*, *FTH1*, *FTL*, *SPP1*). Similar to our coarse-grained microglial comparison, we compared the strength of our approach in astrocytes. After setting significance cutoffs based on our per-gene *q*-values, we identified significantly fewer enriched genes (221, 271, and 886) than we found with our analysis (1444, 680, and 2278 genes for AMD, AD, and MS, respectively) (Supplementary Fig. 10b). In our cell-type level analysis, only 28 common genes were found, significantly less than the 630 common genes found with our pipeline. Of the common genes, only half of the activation signature was found (*AQP4*, *CD81*, *CRYAB*, *GFAP*).

Collectively, these comparisons reveal the sensitivity of this discovery pipeline for finding gene signatures and biologically meaningful relationships in noisy single-cell gene expression data.

### Other computational methods details

#### Single-nucleus AMD RNA sequencing and pre-processing

snRNA-seq data from macular samples, were processed according to the following steps. Sample demultiplexing and read alignment to the NCBI reference pre-mRNA GRCh38 was completed to map reads to both unspliced pre-mRNA and mature mRNA transcripts using CellRanger version 3.1.0. Gene and cell matrices from retinas with dry AMD (*n* = 4), neovascular AMD (*n* = 7), or controls with no known retinal disease (*n* = 6) were then combined into a single file. We prefiltered using parameters in scprep (v1.0.3, https://github.com/KrishnaswamyLab/scprep). Cells that contained at least 1400 unique transcripts were kept for further analysis to generate a cell by gene matrix containing 70,973 cells. Normalization was performed using default parameters with L1 normalization, adjusting total library side of each cell to 1000. Any cell with greater than 200 normalized counts of mitochondrial mRNA was removed. Batch correction was performed using Harmony (https://github.com/immunogenomics/harmony) to align batch effects introduced by sequencing batch, postmortem interval, sample acquisition location and 10X sequencing chemistry^83^. Raw data files for human snRNA-seq data will be available for download through GEO under an accession number to be assigned with no restrictions on data availability.

#### Single-nucleus AD and MS RNA-sequencing pre-processing

snRNA-seq data for AD and MS was acquired from published sources^4,5^. Cells that contained at least 1000 unique transcripts were kept for further analysis to generate a cell by gene matrix for each disease. Normalization was performed using scprep default parameters with L1 normalization, adjusting total library side of each cell to 1000. Any cell with greater than 200 normalized counts of mitochondrial mRNA was removed. Batch correction was performed on MS data using Harmony (https://github.com/immunogenomics/harmony) to align batch effects introduced by sequencing batch, capture batch and sex.

#### Cell-type identification with CATCH

All cell types were identified by performing topological activity analysis on the diffusion condensation calculated condensation homology. In order to identify cell types, we identified a resolutions with no topological activity, which partitioned the cellular state space well and assigned each cluster to a cell type based on cell-type-specific marker genes.

#### Interaction analysis

Cell–cell ligand-receptor analysis was conducted on pre-processed snRNA expression data using the CellPhoneDB python package (https://github.com/Teichlab/cellphonedb, v2.1.4)^52^. Before conducting analysis, the package database of 834 curated ligand-receptor combinations and multi-unit protein complexes was supplemented with 2557 ligand-receptor interactions found in the celltalker database (https://github.com/arc85/celltalker)^84^. The in-built database-generate function was utilized to update the existing database. Our comprehensive user-generated database was invoked in each run of the CellPhoneDB statistical-analysis command function.

CellPhoneDB interaction maps were computed on differing inputs. First, disease-phase enriched microglia and astrocytes with subcluster identity were run to identify signaling interactions between astrocyte and microglial activation states (Fig. 6B). The number of permutations was set to 2000 and *p*-value threshold was set to 0.01.

### Biological methods details

#### Human tissues

Postmortem eyes for the Chromium Single Cell 3’ assay (*n* = 17) and medical records containing AMD disease stage were obtained from Advancing Sight Network (Alabama), Lions Gift of Sight Eye Bank (Minnesota), or the Yale Department of Pathology with a maximum postmortem interval of 13 h. Globes were examined for retinal disease by an ophthalmologist (B.P.H.) prior to dissection and dissociation of the samples. Retina for snRNA-seq was obtained from the unrelated human postmortem donors that included normal, intermediate dry on AREDS2, and neovascular AMD stages (Supplementary Table 1). For each sample we profiled the macula, which is the region of the retina responsible for central vision and affected most severely by AMD pathology. We identified  four intermediate AMD samples from patients taking the AREDS2 eye vitamin and mineral supplement with drusen, a pathologic sign associated with the intermediate dry stage of the disease. Seven postmortem AMD samples had neovascularization in the advanced stage of the disease. Normal donors had no history of retinal disease. Additional clinical data for the subjects is given in Supplementary Table 2.

#### Retinal dissection and isolation of nuclei from frozen retinal tissue

Globes were placed in RNAlater (ThermoFisher) and transported on ice. Trephine punches (6 mm diameter) were used to isolate samples from the macula in the central retina, located away from the optic disc and major arterioles. For each punch of tissue, the retina was mechanically separated from the underlying retinal pigment epithelium-choroid, snap-frozen on dry ice and stored at –80 °C. Nuclei were isolated and purified using the Nuclei EZ Prep Nuclei Isolation Kit (Sigma), following the manufacturer’s protocol, with some modification. All procedures were carried out on ice or at 4°C. Briefly, frozen retinal tissue was subjected to dounce homogenization (25 times with pestle A followed by 25 times with tight pestle B) using the KIMBLE Dounce Tissue Grinder Set (Sigma) in 2 mL EZ Lysis buffer. The sample was transferred to a 15 ml tube with an additional 2 mL EZ lysis buffer and incubated on ice for 5 min. Following incubation, the sample was centrifuged at 500 x *g*, 5 min at 4°C. Supernatants were discarded, and the isolated nuclei were resuspended in 4 mL EZ lysis buffer, incubated for 5min on ice and centrifuged at 500 x *g* for 5 min at 4 °C. Next, the nuclei were washed with 4 mL ice-cold Nuclei Suspension Buffer (1x phosphate-buffered saline (PBS) containing 0.01% BSA and 0.1% RNase inhibitor), resuspended in 1 mL Nuclei EZ Storage buffer and passed through a 40 μM nylon cell strainer. The nuclei suspensions were counted with trypan blue prior to loading on the microfluidics platform.

#### Droplet-based microfluidics snRNA-seq

Isolated nuclei from each macular sample were processed through microfluidics-based single nuclear RNA-seq. Single-cell libraries were prepared using the Chromium 3’ v2 and v3 platforms (10x Genomics) following the manufacturer’s protocol. Briefly, single nuclei were partitioned into Gel beads in Emulsion in the 10x Chromium Controller instrument followed by lysis and barcoded reverse transcription of RNA, amplification, shearing and 5’ adapter and sample index attachment. On average, 7000 nuclei were loaded on each channel that resulted in the recovery of 4000 nuclei. Libraries were sequenced on the Illumina NextSeq 500 platform. Raw sequence data was aligned to GRCh38-3.0.0 human genome using STAR aligner, and Cell Ranger software (v3.1.0, 10x Genomics) was used to demultiplex reads and assign read counts to individual cells. (After quality control pre-processing, snRNA-seq profiles were used in subsequent analyses. This dataset was corrected for batch effects across samples using the Harmony algorithm^83^.

#### In situ RNA hybridization and immunofluorescence

To validate the gene expression differences, in situ hybridization was performed using RNAscope Multiplex Fluorescent V2 Assay (Advanced Cell Diagnostics, Hayward, CA, USA). Macula dissected from whole human globes were fixed in 4% paraformaldehyde (PFA) at 4°C overnight. Tissues were sequentially dehydrated with 15% sucrose, then 30% sucrose before embedding in OCT, and frozen on dry ice. OCT molds were sectioned at 10 μm thickness. RNA in situ hybridization was performed according to the manufacturer’s protocol. Briefly, fixed frozen sections were baked at 60°C for 1 h prior to incubation in 4% PFA for 10 min and protease digestion pretreatment. Target probes were hybridized to an HRP-based temperature sensitive signal amplification system, followed by color development. Housekeeping genes *POLR2A, PPIB*, and *UBC* were used as internal-control mRNA (Supplementary Fig. 7); if probes for these mRNAs were not visualized, the sample was regarded as not available for gene expression study. The probes used include *APOE, TYROBP, B2M, VEGFA*, and *HIF1A* (Advanced Cell Diagnostics, Hayward, CA, USA). The slides were counterstained with DAPI during immunofluorescence protocol (see below). Positive staining was determined by fluorescent punctate dots in the appropriate channels in the nucleus and/or cytoplasm. Following RNA in situ hybridization protocol, fixed frozen sections were blocked with animal serum and incubated overnight at 4°C with primary antibodies (see antibody segment below). Secondary antibody incubation was for 1 h at room temperature and cell nuclei were counterstained with DAPI. Images were captured immediately using a confocal microscope (Zeiss LSM800, Jena, Germany). The following antibodies against human antigens were used: GFAP (1:500, MA5-12023, Invitrogen) and Iba1 (1:500, 019-19741, Fujifilm). Antibodies were visualized with Alexa Fluor 488 (1:200, A-11001/A-21208, Invitrogen).

#### Mice

Four- to 8-week-old mixed sex C57BL/6 mice were purchased from the National Cancer Institute and subsequently bred and housed at Yale University. All procedures used in this study (sex-matched, age-matched) complied with federal guidelines and the institutional policies of the Yale School of Medicine Animal Care and Use Committee (IACUC approved protocol #2022-20275) governing animal welfare and ethical treatment.

#### Cells

IPSC-derived astrocyte cells were purchased from Brainxell.com (Catalog number BX-0600; Brainxell, Madison Wisconsin). Cells were cultured according to provider’s guidelines using 1:1 DMEM/F12 and Neurobasal medium with N2 supplement (1x), Glutamax (0.5mM), Astrocyte supplement (1x), Fetal bovine serum (1%).

#### Cell culture

IPSC-derived astrocyte cells were cultured to a fully differentiated state before cytokine stimulation. Cytokines, (IL-1β, IL2, IL4, IL6, IL7, IL10, IL12, IL15, IL17, IL22, IL23, IFNG, TNF) were all purchased from PeproTech.com (Peprotech, Cranbury, NJ). For single cytokine stimulation, cells were stimulated with each cytokine at a concentration of 100 ng/mL for 24 h. For combinatorial cytokine stimulation, cocktail of all cytokines minus cytokine of interest was made with each cytokine concentration at 50 ng/mL. Cells were stimulated for 24 h before media was collected. Collected media was centrifuged at 1000 x *g* to remove any cells and debris before performing an ELISA.

#### Enzyme-linked immunosorbent assay

Enzyme-linked immunosorbent assay (ELISA) was performed using a mouse VEGF-A ELISA Kit (Cusabio LLC) following the manufacturer’s instructions. Briefly, two wells in a PVC microtiter plate were coated with 100 μL of antigen (10 μg/mL in PBS), after which the plate was sealed and incubated for 2 h at room temperature. Following three washes with PBS and application of blocking buffer (5% dry milk in PBS) the plate was resealed and incubated for 2 h a room temperature. The plate was washed twice with PBS, and anti-VEGF-A antibody in blocking buffer was added to the wells. After another incubation for 2 h at room temperature, the plate was washed 5 times with PBS and 100 μL of the substrate solution was added to the wells. Stop solution was added to the wells and absorbance at 450 nm was recorded in a plate reader.

#### Intravitreal injection

Mice were anaesthetized using a mixture of ketamine (50 mg/kg) and xylazine (5 mg/kg), injected intraperitoneally. Mice eyes were sterilized using betadine. A small hole was made at the lateral aspect of the limbus was made using a 33 gauge insulin syringe. Using a blunt end Hamilton syringe, 1 μL of PBS or IL-1β (100 ng) was injected at a 45 degree angle at the limbus intravitreally. Once the infusion was finished, syringe was left in place for a minute before removal of the syringe. Injection site was washed with sterile PBS and puralube vet ointment was applied to the eyes. Mice were monitored until full recovery.

#### Mice tissue processing and microscopy

Retinas were dissected, fixed in 2% PFA for 1 h and immediately processed in a blocking solution (10% normal donkey serum, 1% bovine serum albumin, 0.3% PBS-Triton X-100) for overnight incubation at 4^∘^C. After incubation, a subset of retinas for RPE imaging were bleached with treatment with 2 mL 30% H_2_O_2_ + 8 mL PBS + 2 NaOH pellets until optically cleared (30 min). Primary antibodies (VEGFA; Invitrogen cat# MA5-13812) were applied and sections were incubated overnight at 4^ ∘^C, then washed five times at room temperature in PBS and 0.5% Triton X-100, before incubation with a fluoro-conjugated secondary antibody diluted in PBS and 0.5% Triton X-100 for 2 h in room temperature. Sections were washed five times at room temperature, stained with DAPI and mounted before imaging. Confocal images were taken on a Leica SP8 microscope. Quantitative analysis was performed using either FIJI or ImageJ image-processing software (NIH or Bethesda) or Imaris 8 software (Oxford Instruments).

#### Statistics and reproducibility

When two independent groups were compared, Welch *t*-test was used when unequal variances were assumed, and Student’s *t*-test for presumed equal variance. All comparisons were made using two-tailed tests. Chi-square tests were used for comparisons of proportions among two groups. In situ hybridization experiments (as represented in Figs. 3G and  4G) were repeated twice in each case. When three or more independent groups were compared, two-sided multinomial tests with multiple comparisons correction was used, where appropriate. Error bars plotted on visualizations of means represent standard error of the mean. Differential expression analysis as part of the CATCH algorithm includes a two-sided Earth Mover’s Distance, i.e., 1-D Wasserstein distance, with significance cutoffs established based on per-gene false-discovery rates (two-sided EMD test with FDR corrected *p*-value < 0.1). In Fig. 3A 141 microgrlia were identified, with 30 found in healthy-enriched population, 32 found in the dry AMD-enriched population and 79 found in the wet neovascular AMD-enriched population. In Fig. 4A, 474 astrocytes were identified with 301 found in the equally proportioned population, 22 found in healthy-enriched population, 96 found in the dry AMD-enriched population and 55 found in the wet neovascular AMD-enriched population.

In Figs. 3f and 4f, the box and whisker plots are defined as follows: the whiskers contain the inner 95% confidence interval, the lower bound of the box is the 25% and upper bound the 75% of values. Finally, median in the center of the box denotes the 50%. In this figure, and below, all values are reported in total normalized gene expression values. In Figure 3, microglial activation signatures are presented in microglia clusters across three neurodegenerative diseases. The AMD control-enriched microglia have a minima signature of 7.3, a lower whisker of 7.5, a lower bound of 7.9, a median of 8.4, an upper bound of 8.8, an upper whisker of 9.0 and a maxima of 9.4. The dry AMD disease-enriched microglia have a minima of 6.6, a lower whisker of 7.0 a lower Bound of 8.3, a median of 9.2, an upper bound of 10.2, an upper whisker of 10.8 and a maxima of 11.9. In the Alzheimer’s disease control-enriched cluster, this signature has a minima of 16.7, a lower whisker of 17.0, a lower bound of 17.2, a median of 17.6, an upper bound of 18.2, an upper whisker of 19.0, and a maxima of 20.1. In the early-disease-enriched cluster, this signature has a minima of 16.3, a lower whisker of 17.7, a lower bound of 20.5, a median of 21.4, an upper bound of 23.2, an upper whisker of 24.2, and a maxima of 25.6. In the early progressive control-enriched MS cluster, this signature has a minima of 11.7, a lower whisker of 12.5, a lower bound of 13.2, a median of 13.8, an upper bound of 15.4, a upper whisker of 17.3 and a maxima of 17.7. In the early progressive disease-enriched cluster, this signature has a minima of 15.4, a lower whisker of 15.5, a lower bound of 17.9, a median of 19.0, an upper bound of 19.9, an upper whisker of 21.4 and a maxima of 21.6. In Fig. 4, astrocyte activation signatures are presented for astrocyte clusters across all three diseases. The AMD control-enriched astrocytes have a minima signature of 1.4, a lower whisker of 1.9, a lower bound of 2.5, a median of 3.0, an upper bound of 3.4, an upper whisker of 4.3 and a maxima of 6.8. The dry AMD disease-enriched astrocytes have a minima of 0.4, a lower whisker of 1.8, a lower bound of 2.5, a median of 3.1, an upper bound of 6.7, an upper whisker of 9.2, and a maxima of 10.8. In the Alzheimer’s disease control-enriched cluster, this signature has a minima of 6.6, a lower whisker of 6.7, a lower bound of 8.8, a median of 9.3, an upper bound of 10.1, an upper whisker of 12.5 and a maxima of 16.8. In the early-disease-enriched cluster, this signature has a minima of 6.4, a lower whisker of 6.5, a lower bound of 9.1, a median of 9.5, an upper bound of 11.3, an upper whisker of 12.4, and a maxima of 14.1. In the early progressive MS control-enriched cluster, this signature has a minima of 3.2, a lower whisker of 3.5, a lower bound of 4.6, a median of 5.3, an upper bound of 6.4, an upper whisker of 7.9, and a maxima of 8.1. In the early progressive disease-enriched cluster, this signature has a minima of 4.3, a lower whisker of 4.4, a lower bound of 6.4, a median of 7.0, an upper bound of 7.8, an upper whisker of 9.4 and a maxima of 14.6.

### Reporting summary

Further information on research design is available in the Nature Portfolio Reporting Summary linked to this article.

## Supplementary information

Supplementary Information

Reporting Summary

## Data Availability

Source data are provided as a Source Data file. Raw and processed data files for the snRNA-seq data used in this study are available for download through GEO under the accession number GSE221042. Data used in this study from ref. ^4^ is available on The Rush Alzheimer’s Disease Center Research Resource Sharing Hub at https://www.radc.rush.edu/docs/omics.htm or at Synapse (https://www.synapse.org/#!Synapse:syn18485175) under the 10.7303/syn18485175. Data used in this study from ref. ^5^ are available in the Sequence Read Archive (SRA) under accession number PRJNA544731 (NCBI Bioproject ID: 544731) or at https://ms.cells.ucsc.edu. Source data are provided with this paper.

## Source data

Source Data

## Code availability

The CATCH package, as implemented in python, is available for download with a guided tutorial on the Krishnaswamy Lab Github page: https://github.com/KrishnaswamyLab/CATCH.
