# Supplementary material for: Single-cell analysis reveals inflammatory interactions driving macular degeneration
Source: Nat Commun. 2023 May 5;14:2589. doi: 10.1038/s41467-023-37025-7 (PMC10162998; doi:10.1038/s41467-023-37025-7)
Supplement: Supplementary file 1 — Supplementary Information [file 41467_2023_37025_MOESM1_ESM.pdf]

Supplementary Figures/Tables

Supplementary Figures

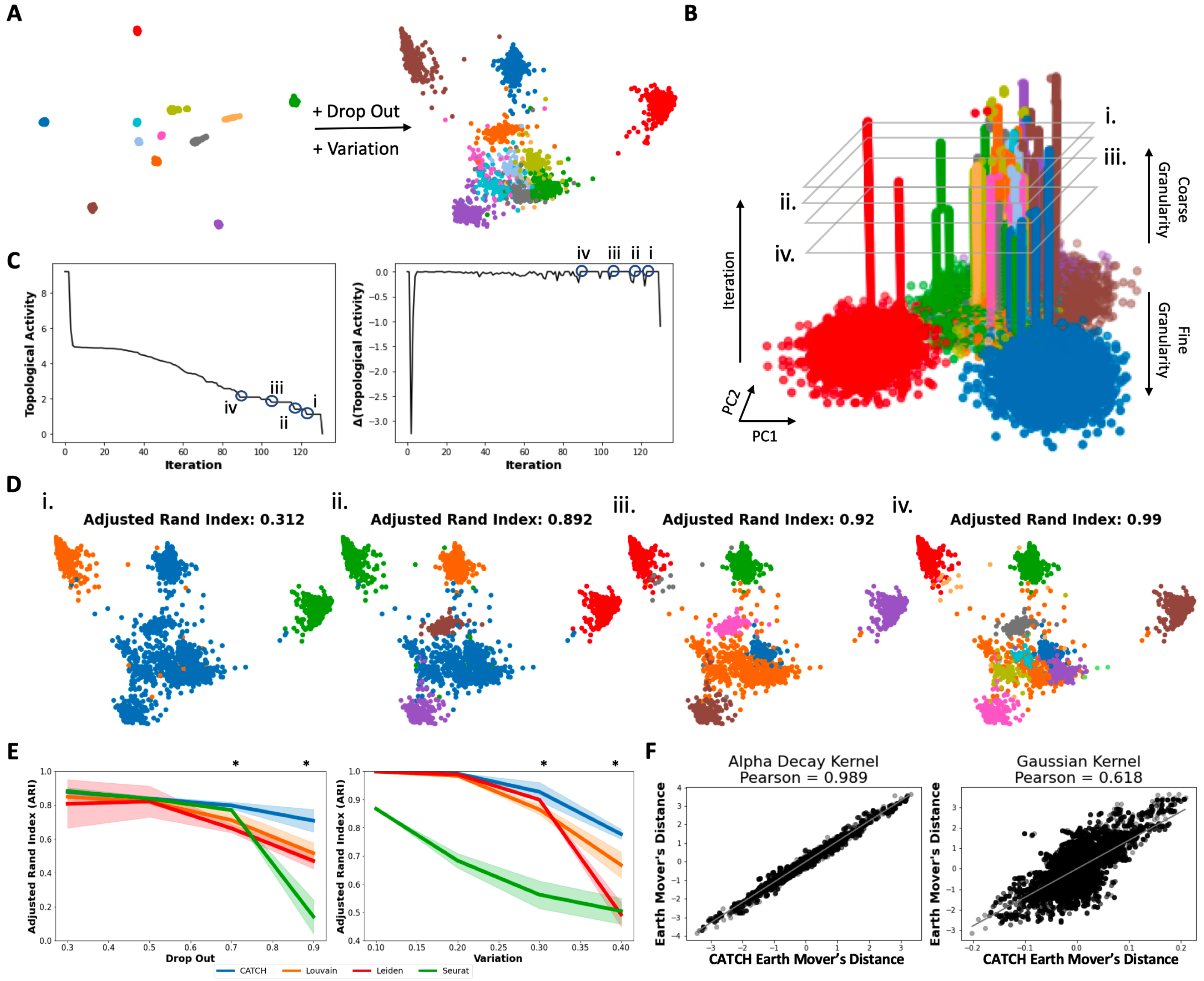

Supplementary Fig. 1: Diffusion condensation identifies and characterizes populations of related cells across granularities. **(A)** PHATE visualization of 10,000 cells generated from splatter [19]. Noiseless data is first generated on which ground truth clusters are computed (left). Two types of biological noise, variation and drop out, are simulated and the dataset is revisualized with ground truth cluster labels highlighted (right). **(B)** condensation homology visualization of noisy splatter single cell data simulation. Four granularities are highlighted (represented as i.-iv.), illustrating 4 resolutions identified in (C) as meaningful. **(C)** Topological activity is first computed (left) before gradient analysis is performed on the topological activity curve (right). Resolutions identified by this analysis as being meaningful are highlighted (represented as i.-iv.). **(D)** Meaningful resolutions identified in (C) are represented with Adjusted Rand Index score when compared to ground truth cluster labels shown. Visualizations are arranged from the coarsest granularity of clusters (left) to the finest granularity (right). **(E)** Forty different splatter synthetic single cell datasets are simulated with either increasing amounts of drop out (left panel) or biological variation (right panel). Cluster labels are computed using a range of multiresolution clustering techniques (CATCH, Louvain, Leiden, Seurat). The top four most optimal resolutions from each algorithm are compared to ground truth cluster labels computed on noiseless data, with the highest Adjusted Rand Index score saved. This is repeated 10 times using different random seeds for each algorithm. Shading represents 2 standard deviations around the mean of each algorithm's performance. At the highest levels of dropout and variational noise, CATCH performs significantly better than Louvain, Leiden and Seurat ($p < 0.05$, ANOVA with post-hoc two-sided Student's t-test with multiple comparisons correction). **(F)** Condensation's approximation Earth Mover's Distance with an α- decay kernel shows superior fidelity with ground truth Earth Mover's Distance on 4,360 single cell PBMCs measured on the 10X platform. The diffusion condensation algorithm was implemented with α- decay (left panel) and Gaussian (right panel) kernels. In each implementation, topological activity analysis identified persistent clustering granularities. For this comparison, granularities with 20 or less clusters were analyzed by comparing all clusters to each other. This resulted in 10,166,640 and 2,541,660 comparisons for the α- decay kernel and Gaussian implementations respectively.

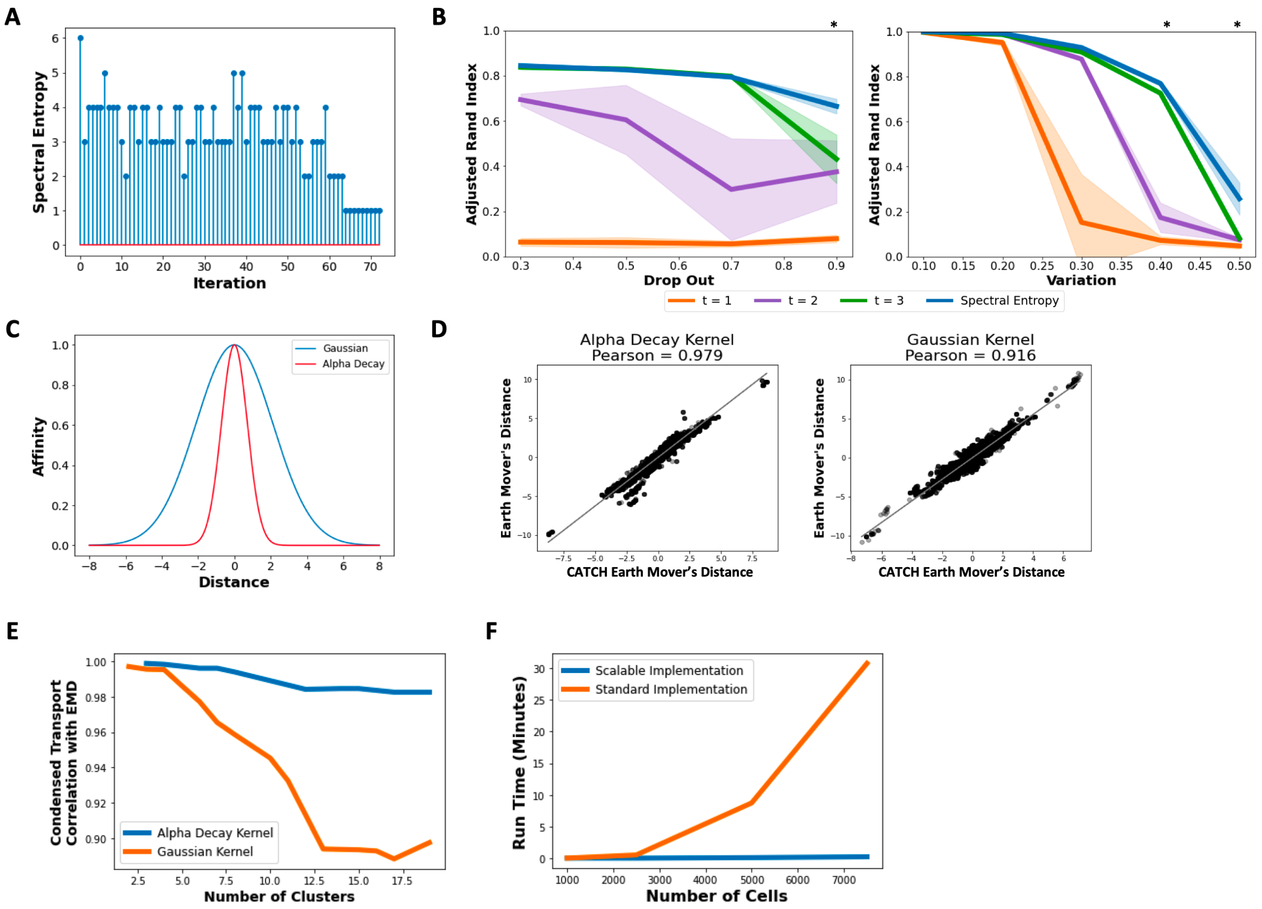

Supplementary Fig. 2: Key advancements in CATCH clustering approach. (A) Ideal number of t-steps calculated by spectral entropy per iteration when running diffusion condensation on 4,360 single cell PBMCs measured on the 10X platform. **(B)** Comparison of different CATCH implementations on synthetic splatter data with increasing levels of noise. Comparing implementations with fixed numbers of t steps set for every iteration (t=1,2,3) against the final diffusion condensation approach which uses spectral entropy to tune t at every iteration. Adaptively tune t outperforms other implementations significantly as noise levels increase across noise types (two-sided Student's t-test, $p < .05$). **(C)** Visualization of difference between Gaussian and α-decay kernels. **(D)** Comparison of differentially expressed genes using CATCH condensed transport implemented with Gaussian kernel and α-decay kernel against ground truth 1D-Wasserstein EMD distance. In each comparison, CATCH was run on 10,000 cells generated from splatter as done in part B. Topological activity analysis was used to compute salient granularities for downstream analysis. In each comparison, all salient granularities with less than 20 clusters was used. At each granularity, we computed differentially expressed genes between every combination of clusters. Across all comparisons, 10,249,140 and 4,535,640 comparisons for the Gaussian and α-decay kernel implementations were computed respectively. **(E)** Comparing correlation between ground truth EMD values with condensed transport values across granularities using the same multi-cluster and multi-granular comparison strategy described in **(D)**. Visualizing reported correlation values as a feature of cluster granularity (denoted by number of cluster on x-axis) for both Gaussian and α-decay kernels. **(F)** Run time comparison between scalable and standard implementations of CATCH.

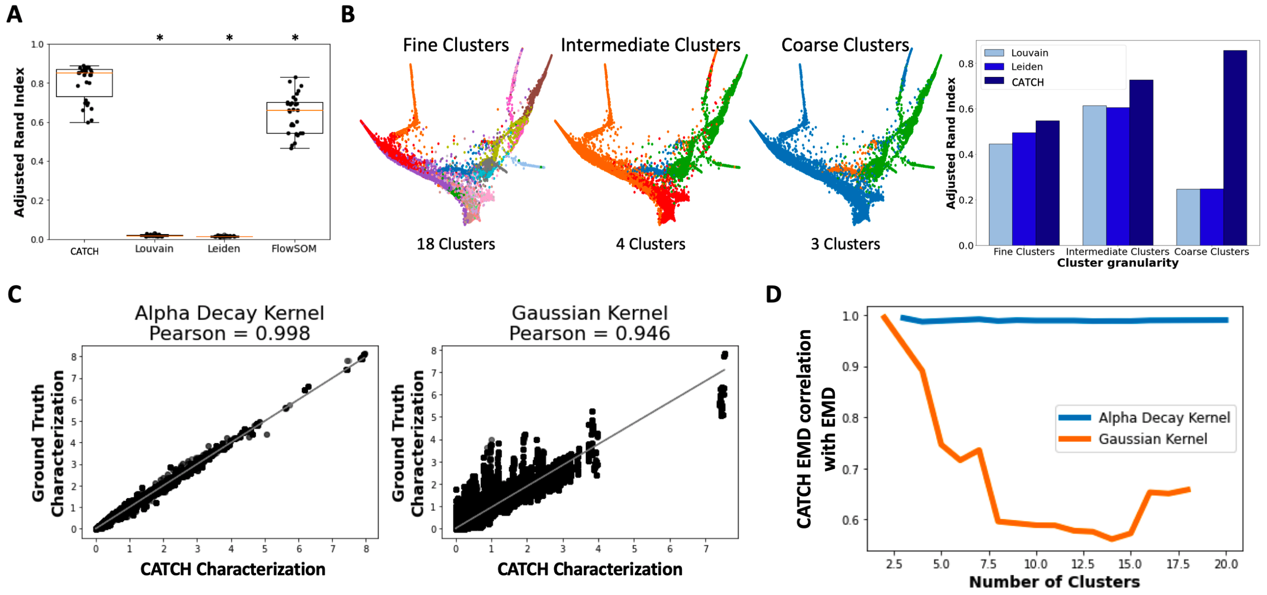

Supplementary Fig. 3: Comparison of diffusion condensation against differing implementations and other clustering approaches on synthetic and real single cell data (A) Comparison of CATCH, Louvain, Leiden and FlowSOM on flow cytometry data with cluster labels have been identified through conventional gating analysis. Comparison was repeated on 1.3 million cells generated from 30 patients in the FlowCAP dataset (center line, median; box limits, upper and lower quartiles; whiskers, 1.5x interquartile range; points, all data; 2 sided T test without multiple hypothesis correction, p-value $< .01$). **(B)** Comparison of CATCH against multigranular clustering approaches, Louvain and Leiden, on real single cell data across clustering granularity. **(C)** Diffusion condensation cluster characterization implemented with α-decay and Gaussian kernels compared to ground truth cluster characterizations across granularities on 4,360 PBMCs measured with 10X. **(D)** Comparing correlation between ground truth EMD values with condensed transport values across granularities using the same multi-cluster and multi-granular comparison strategy described in S1F on 10X single-cell data. Reported correlation values as a feature of cluster granularity (denoted by number of cluster on x-axis) for both Gaussian and α-decay kernels, representing 12,061,332 and 3,373,476 comparisons respectively.

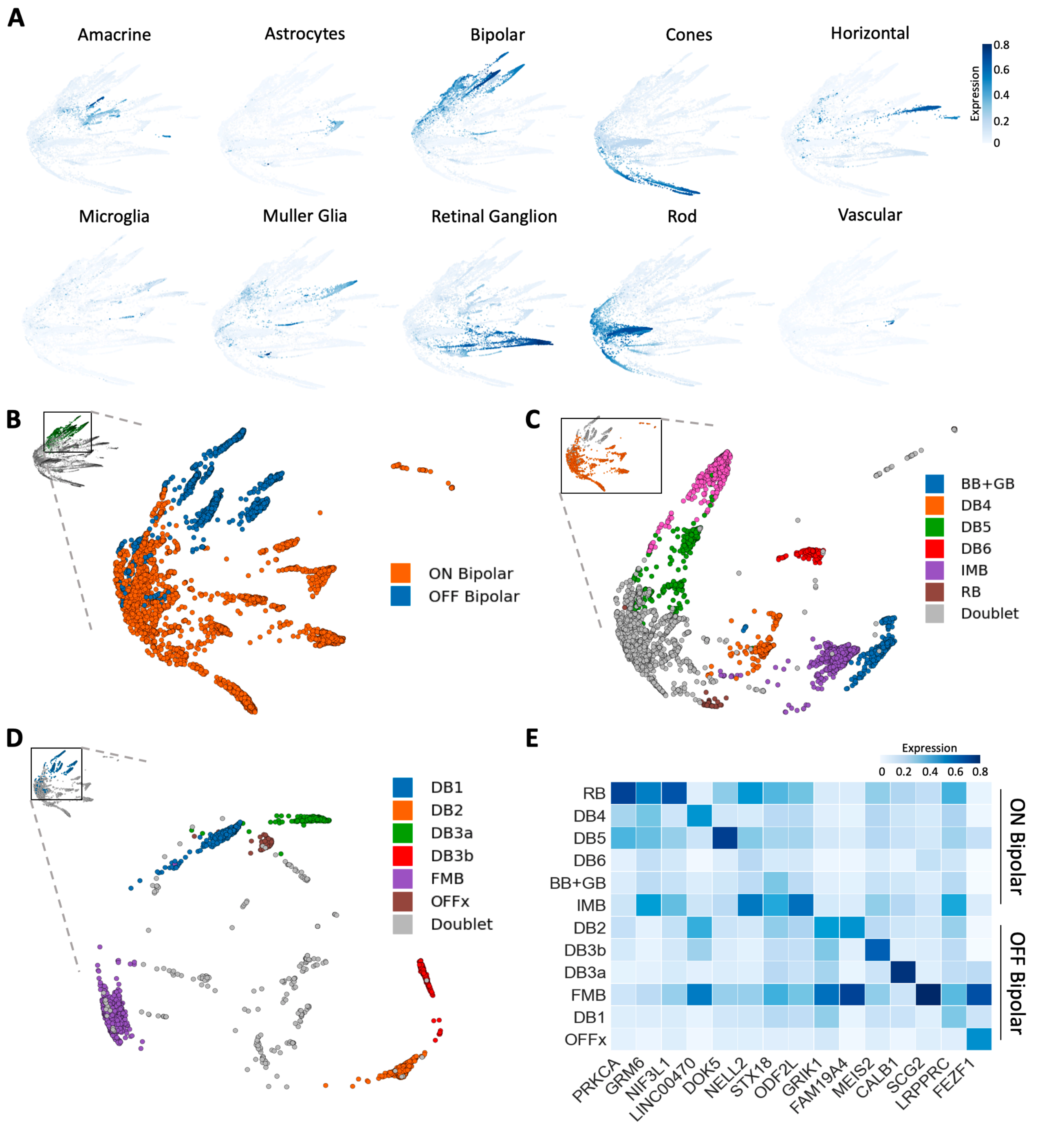

Supplementary Fig. 4: CATCH identifies known subtypes of bipolar cells across multiple levels of granularity. (A) Cell type specific signatures based on composite normalized expression of cell type specific marker genes visualized on PHATE. **(B)** CATCH identifies ON and OFF bipolar cell subsets at a granularity identified with topological activity analysis. **(C)** Finer grained analysis of ON bipolar cells reveals known subsets. **(D)** Finer grained analysis of OFF bipolar cells reveals known subsets. **(E)** CATCH reliably identifies established cell types, as shown by average normalized expression of known bipolar subset-specific marker genes.

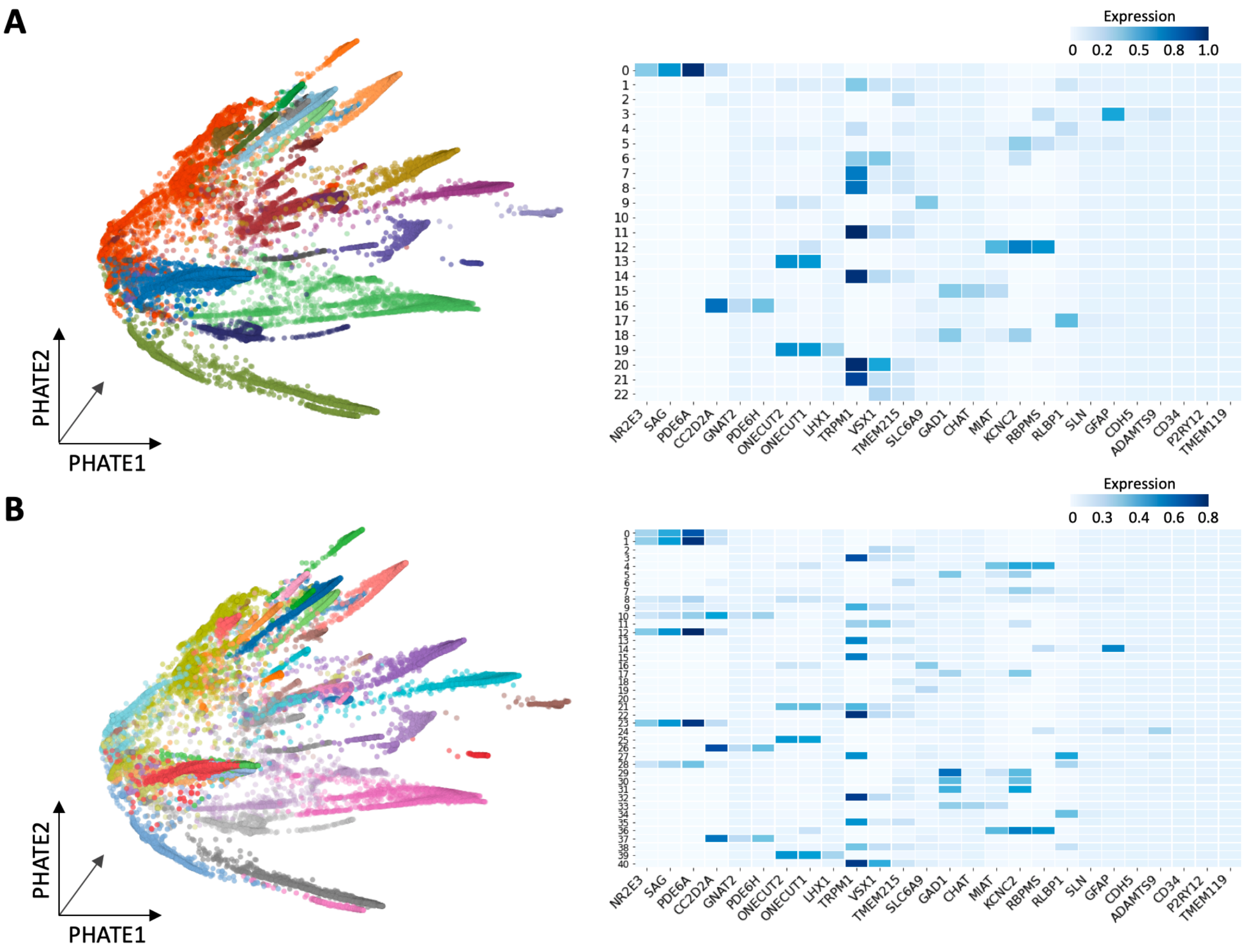

Supplementary Fig. 5: Louvain does not identify rare glial populations across granularities. (A) Visualization of 22 coarse grain clusters identified by Louvain. The identified populations are not able to identify all known cell types, as shown by the average normalized expression of known cell type specific marker genes. **(b)** Visualization of 40 fine grain clusters identified by Louvain. The identified populations at this granularity also do not resolve all known cell types, as shown by the average normalized expression of known cell type specific marker genes.

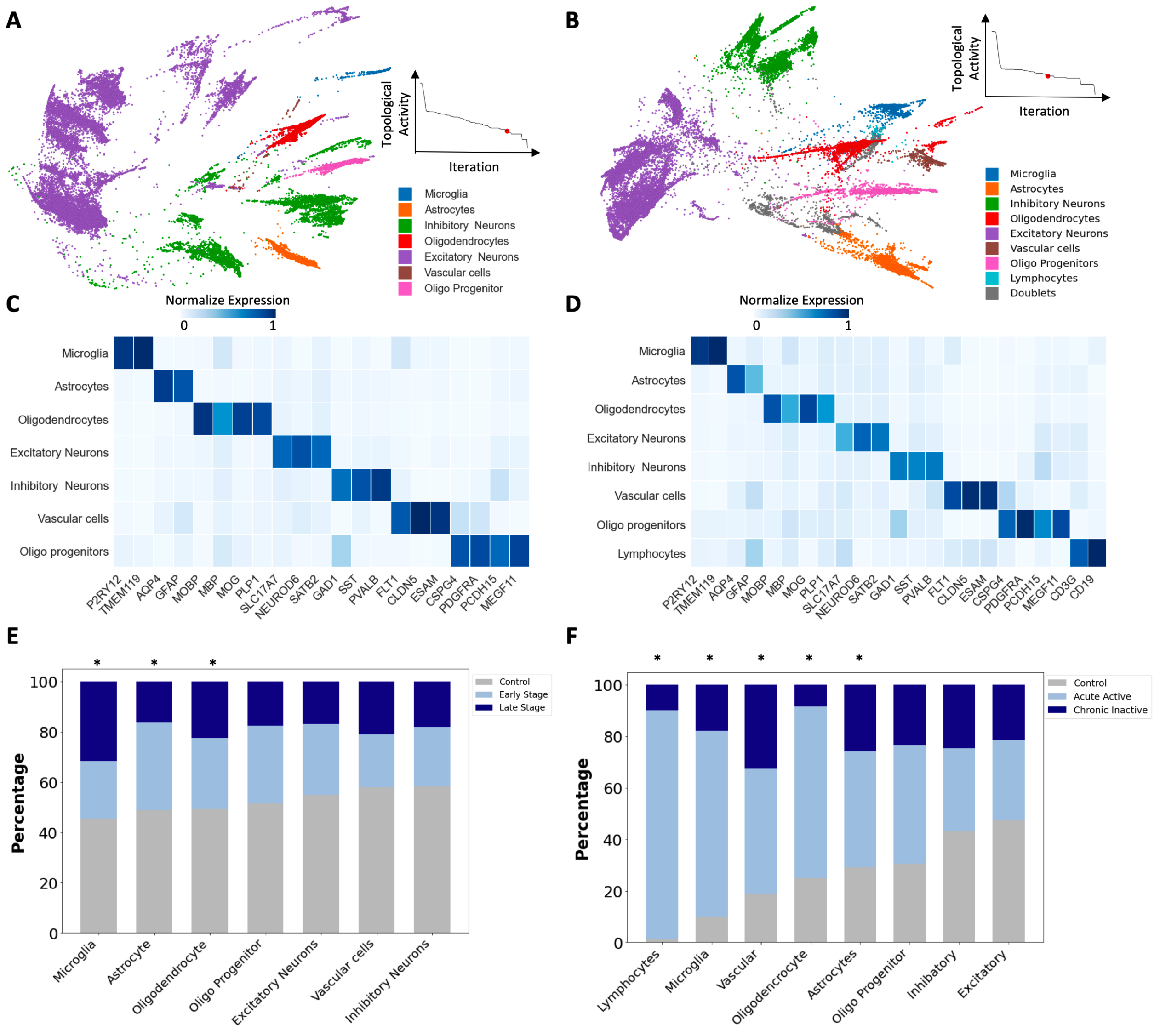

Supplementary Fig. 6:

CATCH analysis of AD and MS snRNAseq data reveals enrichment and activation of microglia and astrocytes in disease. **(A)** 43,650 cells pooled from 48 AD patients and healthy donors. Samples were taken from disease free brain tissue and diseased brain tissue at early and late pathological stages. All major cell types were identified by CATCH via persistence analysis and visualized with PHATE [65]. Ideal CATCH granularity identified via topological activity analysis (right). **(B)** 46,796 cells pooled from 21 progressive MS patients and healthy donors. Samples were taken from disease free brain tissue and diseased brain tissue at acute and chronic stages of inflammation. All major cell types were identified by CATCH via persistence analysis and visualized with PHATE [65]. Ideal CATCH granularity identified via topological activity analysis (right). **(C)** CATCH reliably identifies cell types in AD brain tissue, as shown by average normalized expression of known cell type-specific marker genes. **(D)** CATCH reliably identifies cell types in MS brain tissue, as shown by average normalized expression of known cell type-specific marker genes. **(E)** Microglia and astrocytes are the most enriched cell types in AD using cross condition abundance analysis. **(F)** Microglia and astrocytes are significantly enriched in progressive MS using cross condition abundance analysis. In E,F: * = $p < 0.01$, two-sided multinomial test with multiple comparisons correction.

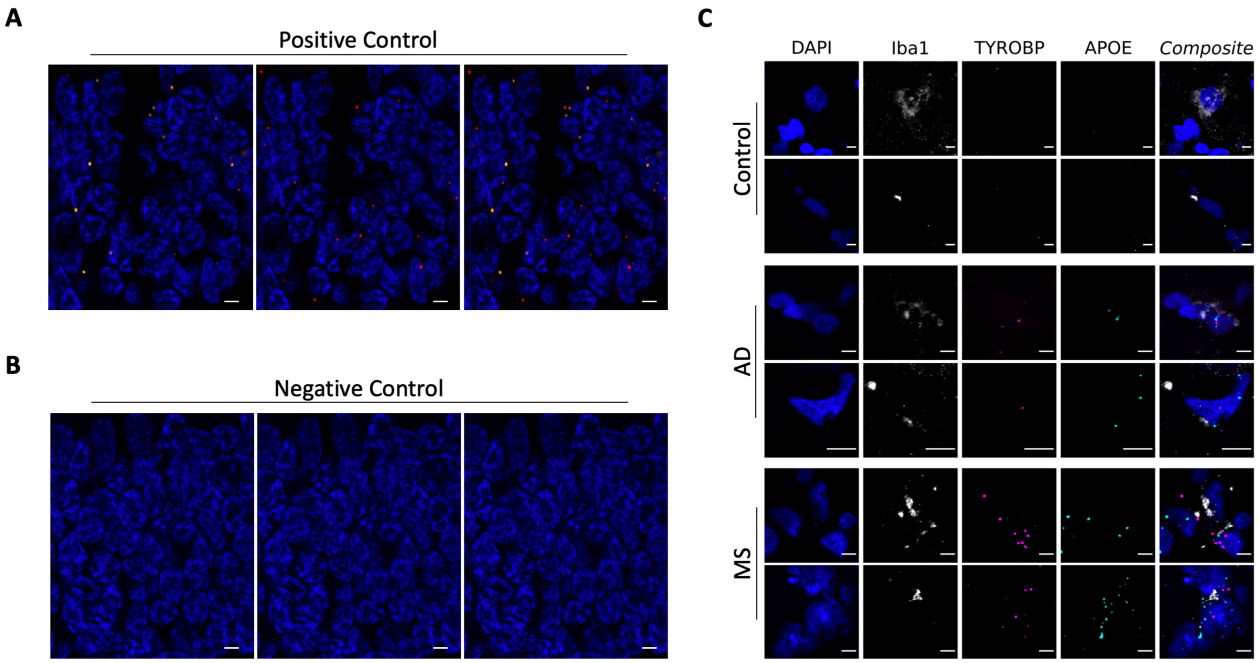

Supplementary Fig. 7: Control probes for fluorescence in situ hybridization and validation of early activation microglial signature in MS and AD. Representative images of fluorescence in situ hybridization for **(A)** positive control probe (POL2RA labeled in red and UBC labeled in yellow) and **(B)** negative control probe (DapB labeled in yellow and red). **(C)** Representative images of in situ RNA hybridization of *APOE* (labeled in turquoise), *TYROBP* (labeled in pink) with simultaneous immunofluorescence of microglial marker IBA1 (white). Elevated expression of *APOE* and *TYROBP* is seen in IBA1-positive cells in microglia from brain tissue with early AD and early progressive MS, compared to healthy controls. Each row represents a sample from a different case. All scale bars = 10µm.

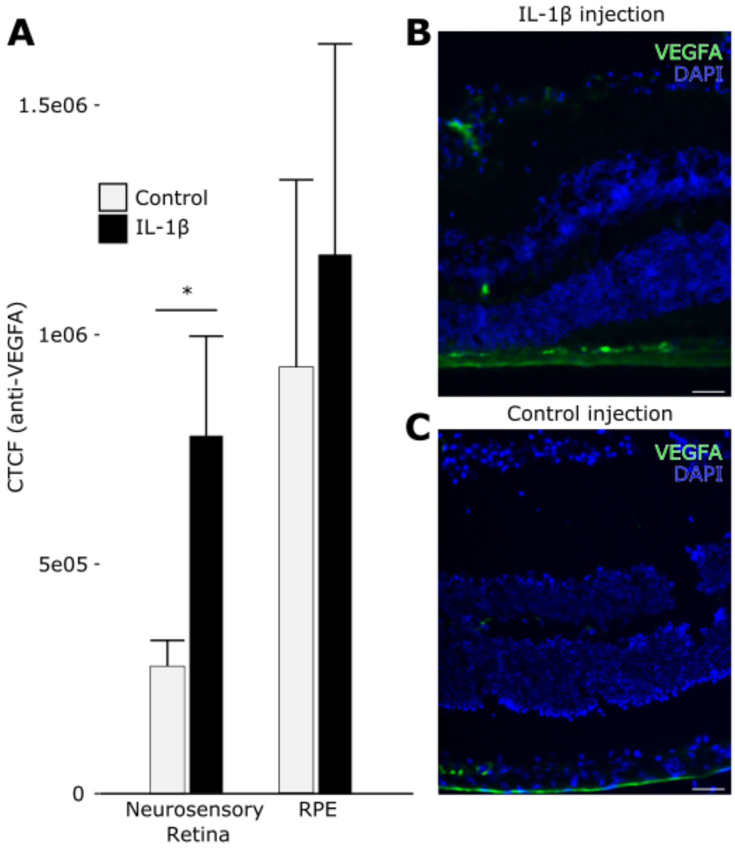

Supplementary Fig. 8: IL-1β injection affects expression of VEGFA in neurosensory retina cells and retinal pigment epithelium. IL-1β (n=4) or PBS (n=4) was injected intravitreally into mouse eyes. Retinas were collected 72 hours later for immunofluorescent imaging of sections stained with anti-VEGFA fluorescent antibody following peroxide bleaching of endogenous pigment. **(A)** Quantification of corrected total cell fluorescence (CTCF) in n=60 manually segmented cells after injection of IL-1β or PBS control in the mouse eyes after 72 hours in cells residing in the neurosensory retina (left) or retinal pigment epithelium (RPE; right). Bar heights represent means; error bars represent standard error of the mean (SEM). * = $p < 0.5$; two-sided student's t-test; Two-way ANOVA revealed no significant differences between neurosensory and RPE VEGFA expression in either condition. **(B)** and **(C)** Example fluorescence micrographs of retinas exposed to IL-1β or PBS control, respectively; scale bars = 50μm.

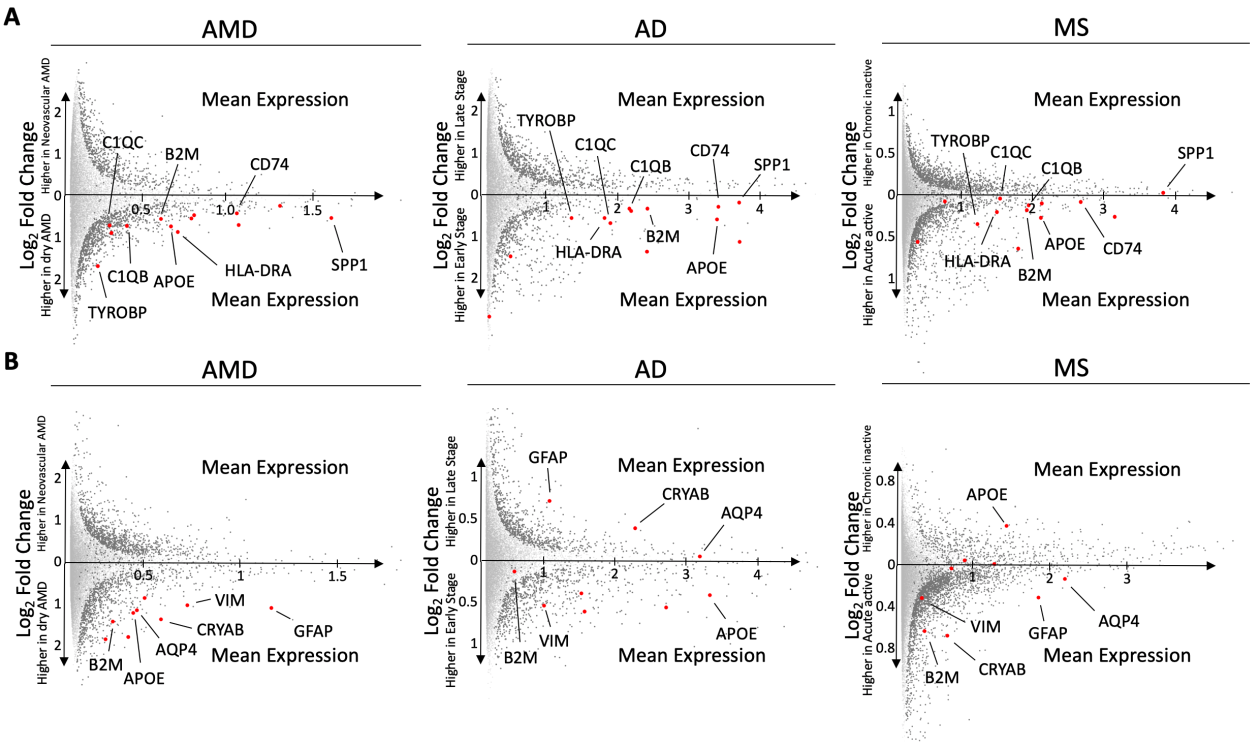

Supplementary Fig. 9: Shared activation signatures is diminished in advanced disease and replaced with disease-specific stress signature in microglia. **(A)** Comparing advanced or chronic inactive disease-enriched microglial cluster to early or acute active-enriched microglial cluster reveals a significant reduction in the microglial activation signature in later stages of neurodegeneration. Significant differentially expressed genes visualized in dark grey (two-sided EMD test with FDR corrected p-value $< .1$ as described in methods) and shared gene signature shown in red. **(B)** Comparing advanced or chronic inactive disease-enriched astrocyte cluster to early or acute active-enriched cluster reveals a significant reduction in the astrocyte activation signature in later stages of neurodegeneration. Significant differentially expressed genes visualized in dark grey (two-sided EMD test with FDR corrected p-value $< .1$ as described in methods) and shared gene signature shown in red.

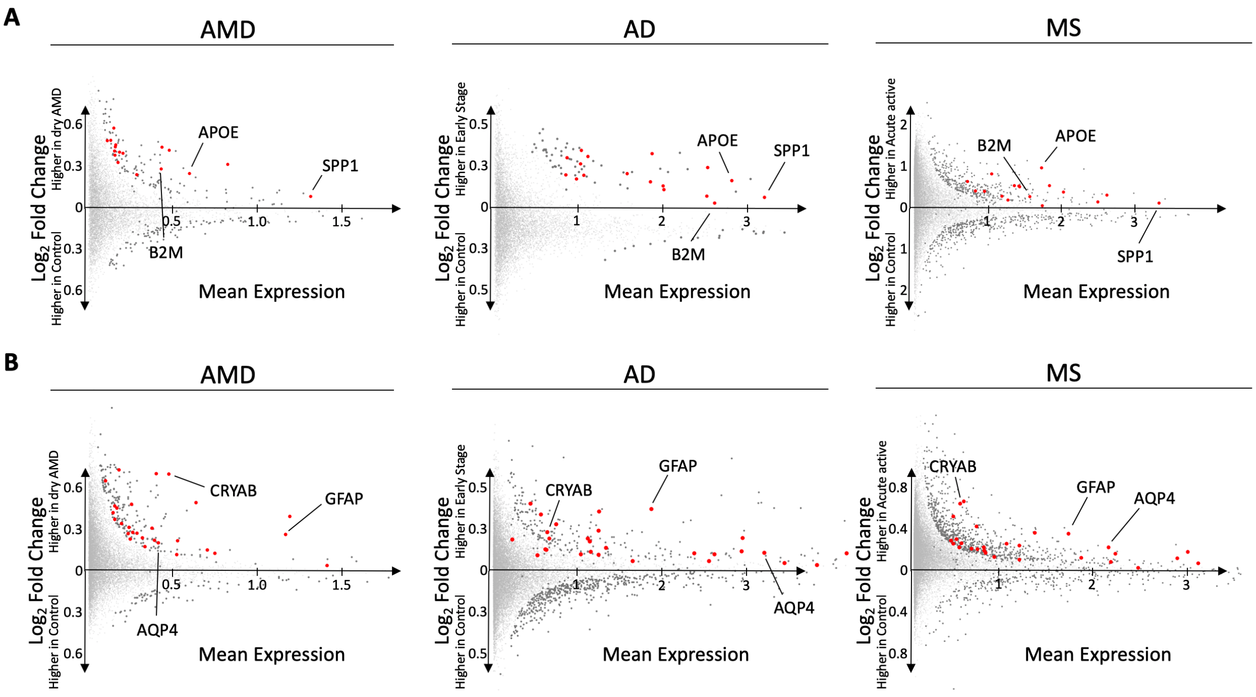

Supplementary Fig. 10: Cell type level differential expression analysis at coarse granularity across neurodegenerative diseases. **(A)** Performing EMD-based differential expression analysis between microglia which originate from dry AMD patients and control subjects identified a gene signature enriched in the early stage of dry AMD. By performing similar differential expression analysis between microglia from brain samples from patients with early AD, acute active MS, and controls, we identified a shared activation signature of 17 genes. This common signature includes *APOE*, *SPP1* and *B2M* while all other genes are highlighted in red. Significant differentially expressed genes visualized in dark grey (two-sided EMD test with FDR corrected p-value $< .1$ as described in methods). **(B)** Performing EMD-based differential expression analysis between astrocytes which originate from dry AMD patients and control subjects identified a gene signature enriched in the early stage of dry AMD. By performing similar differential expression analysis between astrocytes from control and early AD samples and control and acute inflammation MS samples, we identify a shared activation signature of 28 genes. This common signature includes *GFAP*, *AQP4*, and *CRYAB* while all other genes are highlighted in red. Significant differentially expressed genes visualized in dark grey (two-sided EMD test with FDR corrected p-value $< .1$ as described in methods).

Supplementary Tables

Retina	Sex	Age	Postmortem Interval (Hrs)	Left/Right Eye	Condition	Assay
1	F	90	2	Right	Control	snRNA-seq
2	F	81	4	Left	Control	snRNA-seq
3	M	65	8	Left	Control	snRNA-seq
4	M	78	4	Left	Control	snRNA-seq
5	F	72	4	Left	Control	snRNA-seq
6	M	85	3	Right	Control	snRNA-seq
7	M	86	9	Left	Intermediate Dry AMD	snRNA-seq
8	M	72	3	Left	Intermediate Dry AMD	snRNA-seq
9	F	82	4	Right	Intermediate Dry AMD	snRNA-seq
10	F	74	5	Right	Intermediate Dry AMD	snRNA-seq
11	F	79	10	Right	Neovascular AMD	snRNA-seq
12	F	100	9	Left	Neovascular AMD	snRNA-seq
13	F	93	8	Right	Neovascular AMD	snRNA-seq
14	F	94	13	Left	Neovascular AMD	snRNA-seq
15	F	92	<1	Right	Neovascular AMD	snRNA-seq
16	F	76	9	Left	Neovascular AMD	snRNA-seq
17	F	67	2	Left	Neovascular AMD	snRNA-seq

Supplementary Table 1: Human retinal specimen details.

Subject	Sex	Age	Smoking History	Hypertension	Diabetes	Treatment anti-VEGF	Treatment AREDS vitamins
1	F	90	No	No	No	No	No
2	F	81	Unk.	Unk.	Unk.	Unk.	Unk.
3	M	65	No	No	No	No	No
4	M	78	No	Yes	No	No	No.
5	F	72	No	No	No	No	No
6	M	85	No	No	No	No	No
7	M	86	Former smoker	No	No	No	No
8	M	72	No	No	No.	No	Yes
9	F	82	No	No	No	No	Yes
10	F	74	No	No	No	Yes	Yes
11	F	79	Unk.	Unk.	Unk.	Unk.	Unk.
12	F	100	Unk.	Unk.	Unk.	Unk.	Unk.
13	F	93	Unk.	Unk.	Unk.	Unk.	Unk.
14	F	94	Unk.	Unk.	Unk.	Unk.	Unk.
15	F	92	Unk.	Unk.	Unk.	Unk.	Unk.
16	F	76	No	Yes	No	Yes	Yes
17	F	67	Yes	Yes	No	Yes	No

Supplementary Table 2: Human subject clinical information. 'Unk.' = Data not present in available records.

Retina	Sex	Age	Postmortem Interval (Hrs)	Cells post QC	UMI/Cell	Genes/Counts
1	F	90	2	3362	6596	2081
2	F	81	4	3019	9107	2369
3	M	65	8	4427	10992	2547
4	M	78	4	1183	5253	1779
5	F	72	4	4086	5923	1839
6	M	85	3	1321	3053	1293
7	M	86	9	7888	5656	1889
8	M	72	3	6318	9579	2711
9	F	82	4	4816	6087	1958
10	F	74	5	5129	5154	1501
11	F	79	10	3466	4989	1631
12	F	100	9	5349	8565	2378
13	F	93	8	4621	8644	2653
14	F	94	13	281	2187	1004
15	F	92	<1	6696	5426	1865
16	F	76	9	7476	6408	2111
17	F	67	2	1535	9605	2601

Supplementary Table 3: Human retinal specimen details.
