# Supplementary material for: Single-cell analysis reveals inflammatory interactions driving macular degeneration
Source: Nat Commun. 2023 May 5;14:2589. doi: 10.1038/s41467-023-37025-7 (PMC10162998; doi:10.1038/s41467-023-37025-7)
Supplement: Supplementary file 2 — Reporting Summary [file 41467_2023_37025_MOESM2_ESM.pdf]

nature portfolio

Corresponding author(s): Brian P. Hafler M.D., Ph.D.
Smita Krishnaswamy Ph.D.

Last updated by author(s): Jan 26, 2023

Reporting Summary

Nature Portfolio wishes to improve the reproducibility of the work that we publish. This form provides structure for consistency and transparency in reporting. For further information on Nature Portfolio policies, see our Editorial Policies and the Editorial Policy Checklist.

Statistics

For all statistical analyses, confirm that the following items are present in the figure legend, table legend, main text, or Methods section.

n/a	Confirmed	
☐	☒	The exact sample size (*n*) for each experimental group/condition, given as a discrete number and unit of measurement
☐	☒	A statement on whether measurements were taken from distinct samples or whether the same sample was measured repeatedly
☐	☒	The statistical test(s) used AND whether they are one- or two-sided<br>*Only common tests should be described solely by name; describe more complex techniques in the Methods section.*
☐	☒	A description of all covariates tested
☐	☒	A description of any assumptions or corrections, such as tests of normality and adjustment for multiple comparisons
☐	☒	A full description of the statistical parameters including central tendency (e.g. means) or other basic estimates (e.g. regression coefficient) AND variation (e.g. standard deviation) or associated estimates of uncertainty (e.g. confidence intervals)
☐	☒	For null hypothesis testing, the test statistic (e.g. *F*, *t*, *r*) with confidence intervals, effect sizes, degrees of freedom and *P* value noted<br>*Give P values as exact values whenever suitable.*
☒	☐	For Bayesian analysis, information on the choice of priors and Markov chain Monte Carlo settings
☒	☐	For hierarchical and complex designs, identification of the appropriate level for tests and full reporting of outcomes
☐	☒	Estimates of effect sizes (e.g. Cohen's *d*, Pearson's *r*), indicating how they were calculated

Our web collection on statistics for biologists contains articles on many of the points above.

Software and code

Policy information about availability of computer code

Data collection	Single-nucleus RNA sequencing data was generated using the Illumina NextSeq 500 according to the manufacture's instructions.
Data analysis	To analyze data, we used publicly available software, Cell Ranger Software (version 3.1.0; 10X Genomics), bcl2fastq2, STAR aligner, Python, MELD, MAGIC, scprep (version 1.0.3), CellPhoneDB (version 2.1.4), PHATE, Diffusion condensation, and ImageJ. Custom code used in computational analyses is available at https://github.com/KrishnaswamyLab/Diffusion_Condensation.

For manuscripts utilizing custom algorithms or software that are central to the research but not yet described in published literature, software must be made available to editors and reviewers. We strongly encourage code deposition in a community repository (e.g. GitHub). See the Nature Portfolio guidelines for submitting code & software for further information.

Data

Policy information about availability of data

All manuscripts must include a data availability statement. This statement should provide the following information, where applicable:

- Accession codes, unique identifiers, or web links for publicly available datasets
- A description of any restrictions on data availability
- For clinical datasets or third party data, please ensure that the statement adheres to our policy

Raw and processed data files for human snRNA-seq data are available for download through GEO under accession number GSE221042.

Human research participants

Policy information about studies involving human research participants and Sex and Gender in Research.

Reporting on sex and gender	Patient sex information was collected as well as considered in study design. Gender information was not collected. This is reported in Supplemental Table 1.
Population characteristics	Patient characteristics were collected in Supplemental Table 1. We designed the study to include both male and female samples including a range in the age of donors from 65-100.
Recruitment	All human tissue samples were obtained with informed consent prior to tissue collection from participants if enrolled antemortem or legal guardians if post-mortem from Advancing Sight Network (Alabama), Lions Gift of Sight Eye Bank (Minnesota), or the Yale Department of Pathology. There was no obvious bias in the selection.
Ethics oversight	This study, acquisition, and use of post-mortem human retinal samples was approved by the Yale Human Research Protection Program's Institutional Review Board (Yale Protocol Number 2000028616).

Note that full information on the approval of the study protocol must also be provided in the manuscript.

Field-specific reporting

Please select the one below that is the best fit for your research. If you are not sure, read the appropriate sections before making your selection.

[x] Life sciences [ ] Behavioural & social sciences [ ] Ecological, evolutionary & environmental sciences

For a reference copy of the document with all sections, see nature.com/documents/nr-reporting-summary-flat.pdf

Life sciences study design

All studies must disclose on these points even when the disclosure is negative.

Sample size	No predetermined sample sizes were performed. We analyzed retinal tissue from at lease three individuals per group. This number of samples was sufficient to confidently annotate individual cell groups, and perform a data analysis.
Data exclusions	As described in methods, retinas collected for this study had no abnormalities indicative of disease pathology other than age-related macular degeneration. Control retinas had no known retinal disease. A permissive control step was performed, followed by downstream analysis using the diffusion condensation framework. Putative low-quality cells were filtered out as needed using parameters in scprep. Cells that contained at least 1400 unique transcripts were kept for further analysis. Any cell with greater than 200 normalized counts of mitochondrial mRNA was removed. Harmony was used for batch correction to remove residual batch effects.
Replication	Verification of the single-nucleus RNA-seq data was performed for a subset of genes using fluorescent RNA in situ hybridization on human retinal tissue.
Randomization	Retinal tissue was not randomized. Covariates were controlled in the single-nucleus RNA-seq analysis by isolating postmortem eyes under 10 hours postmortem interval so that the samples are matched.
Blinding	The investigators were blinded during quantifications of the fluorescence in situ hybridization analysis. The single-nucleus RNA-sequuencing analysis was not blinded.

Reporting for specific materials, systems and methods

We require information from authors about some types of materials, experimental systems and methods used in many studies. Here, indicate whether each material, system or method listed is relevant to your study. If you are not sure if a list item applies to your research, read the appropriate section before selecting a response.

Materials & experimental systems

n/a	Involved in the study	
[ ]	[x]	Antibodies
[x]	[ ]	Eukaryotic cell lines
[x]	[ ]	Palaeontology and archaeology
[ ]	[x]	Animals and other organisms
[x]	[ ]	Clinical data
[x]	[ ]	Dual use research of concern

Methods

n/a	Involved in the study	
[x]	[ ]	ChIP-seq
[x]	[ ]	Flow cytometry
[x]	[ ]	MRI-based neuroimaging

Antibodies

Antibodies used	GFAP (1:500, MA5-12023, Invitrogen) and Iba1 (1:500, 019-19741, Fujifilm). Antibodies were visualized with Alexa Fluor 488 (1:200, A-11001 / A-21208, Invitrogen).
Validation	The GFAP and IBA antibodies have been described and validated previously in publications, as well as by the company for IHC.

Animals and other research organisms

Policy information about studies involving animals; ARRIVE guidelines recommended for reporting animal research, and Sex and Gender in Research

Laboratory animals	Four to eight week-old mixed sex C57BL/6 mice were used.
Wild animals	No wild animals were used in this study.
Reporting on sex	Both sexes (males and females) were used in this study.
Field-collected samples	No field-collected samples were used in this study.
Ethics oversight	All procedures used in this study complied with federal guidelines and the institutional policies of the Yale School of Medicine Animal Care and Use Committee (IACUC approved protocol #2022-20275)

Note that full information on the approval of the study protocol must also be provided in the manuscript.
